# Complex viral interactions revealed for the harmful bloom-forming dinoflagellate *Karenia brevis*

**DOI:** 10.1093/ismeco/ycag051

**Published:** 2026-03-09

**Authors:** Anne E Booker, Cong Fei, Shady A Amin, James Custer, Kai Watkins, William Yaeger, So Hyun Ahn, Nayani K Vidyarathna, Alexandra Burns, Sarah Klass, Patricia M Glibert, Cynthia A Heil, Frederik Schulz, Joaquín Martínez Martínez

**Affiliations:** Horn Point Laboratory, University of Maryland Center for Environmental Science, Cambridge, MD 21613, United States; Marine Microbiomics Laboratory, Biology Program, New York University Abu Dhabi, Abu Dhabi 129188, UAE; Marine Microbiomics Laboratory, Biology Program, New York University Abu Dhabi, Abu Dhabi 129188, UAE; Mubadala ACCESS Center, New York University Abu Dhabi, Abu Dhabi 129188, UAE; School of Marine and Environmental Programs, University of New England, Biddeford, ME 04005, United States; School of Marine and Environmental Programs, University of New England, Biddeford, ME 04005, United States; Department of Ecology & Evolution, University of Chicago, Chicago, IL 60637, United States; Biology Department, Woods Hole Oceanographic Institution, Woods Hole, MA 02543, United States; Horn Point Laboratory, University of Maryland Center for Environmental Science, Cambridge, MD 21613, United States; Horn Point Laboratory, University of Maryland Center for Environmental Science, Cambridge, MD 21613, United States; Mote Marine Laboratory, Mote Red Tide Institute, Sarasota, FL 34236, United States; Horn Point Laboratory, University of Maryland Center for Environmental Science, Cambridge, MD 21613, United States; Mote Marine Laboratory, Mote Red Tide Institute, Sarasota, FL 34236, United States; DOE Joint Genome Institute, Lawrence Berkeley National Laboratory, CA 94720, United States; Horn Point Laboratory, University of Maryland Center for Environmental Science, Cambridge, MD 21613, United States

**Keywords:** giant viruses, harmful algal blooms, polinton-like viruses, virus-host linkages, red tide, West Florida shelf

## Abstract

*Karenia brevis* regularly forms harmful blooms along the West Florida Shelf that negatively affect marine and terrestrial organisms through toxin production. These blooms impose economic and environmental hardship, driving the need for research to understand the factors influencing their dynamics and to mitigate their impacts. A mostly unresolved issue is the potential role of viruses in bloom termination. We conducted an experiment incubating *K. brevis* cultures with size-fractionated bloom water samples. Flow cytometry revealed giant virus-like populations (VLPs) in replicate cultures with <1 μm-filtered and <0.2 μm-filtered bloom water. The VLPs’ abundance was paralleled by declines in photoefficiency and culture lysis. Metagenomic analyses of the lysates revealed 11 giant virus genomes (35%–100% complete) representing 7 viral operational taxonomic units (vOTUs) within the order *Imitervirales* (*Nucleocytoviricota*). Ten of these vOTUs were more abundant in the incubations with <0.2 μm-filtered bloom water, coinciding with the absence or low abundance of algicidal bacteria. The vOTUs and *K. brevis* cell abundances showed a positive correlation at a coastal site during bloom and nonbloom periods. The most apparent association was to vOTU6, which may owe its competitive advantage to the presence of the auxiliary metabolic genes bacteriorhodopsin, carbonic anhydrase, and dinoflagellate viral nucleoprotein. The metagenomes also contained polinton-like virus (PLV) genomes. Since many PLVs are hypothesized to depend on co-infection with *Nucleocytoviricota* viruses for their propagation, our results suggest complex viral interactions within *K. brevis* blooms. Future research to elucidate virus–bacteria–*K. brevis* interaction mechanisms may be key to understanding bloom dynamics and developing management tools.

## Introduction

Despite phytoplankton’s beneficial roles at the base of aquatic food webs, as food and removing atmospheric CO_2_, harmful algal blooms (HABs) are detrimental, particularly those of toxin-producing species [1]. HABs are increasing in size and frequency worldwide, likely due to anthropogenic activities leading to eutrophication and climate change [2–4]. As these trends continue, advancing our understanding of factors influencing bloom initiation, maintenance, and termination is critical to aid local stakeholders in developing management and mitigation tools. The mixotrophic dinoflagellate *Karenia brevis* (Alveolata) forms dense blooms along the west coast of Florida almost every year, unpredictably lasting from 3 to 17 months [5, 6]. These blooms produce lethal concentrations of neurotoxic brevetoxins [7] that affect aquatic wildlife by targeting their nervous system [8, 9] and induce respiratory distress in land vertebrates, including humans [10]. The devastating ecological and economic consequences of *K. brevis* blooms [11, 12] make them a target of extensive research and monitoring efforts aimed at understanding their causes and dynamics, with stakeholders particularly interested in understanding termination factors [13].

The combination of physical currents and nutrient supply exerts a strong control on *K. brevis* blooms phases, from initiation to termination [5, 14]. Biological drivers, namely mixotrophy and bacterial interactions (beneficial and detrimental), may also be critical bloom controls [15–19]. However, data on viral infection of *K. brevis* are scant, despite a handful of examples of viral interactions with other bloom-forming toxigenic dinoflagellates. Small RNA viruses are lytic to the bivalve-killing dinoflagellate *Heterocapsa circularisquama* [20]. *Heterocapsa circularisquama* and *H. pygmaea* infection by giant dsDNA viruses in the phylum *Nucleocytoviricota* seems to influence bloom termination [21–23], while evidence of giant virus chronic infection was found during a *Prorocentrum shikokuense* toxic bloom with no change in the host population numbers [24].

In the early 2000s, it was proposed that bacteriophage lysis of cocultured beneficial bacteria caused *K. brevis* cell death [25]. This hypothesis was supported by a multiyear study of *K. brevis* blooms, which found that bacterial concentrations decreased as viral concentrations increased during late bloom stages [26]. Recently, metagenomic analyses have revealed the presence of ssRNA viruses that positively correlate with *K. brevis* concentrations on the West Florida Shelf [27]. Given the growing anecdotal evidence that marine viruses influence *K. brevis* bloom dynamics, we performed an experiment in which *K. brevis* strain CCMP2228 cultures were incubated with size-fractionated seawater from a coastal bloom event. This approach aimed to enrich for *K. brevis* viruses and further elucidate the composition of the viral community associated with *K. brevis* and how virus replication may impact bloom dynamics.

## Materials and methods

### Isolation of *Karenia brevis* viruses

**Figure 1:**
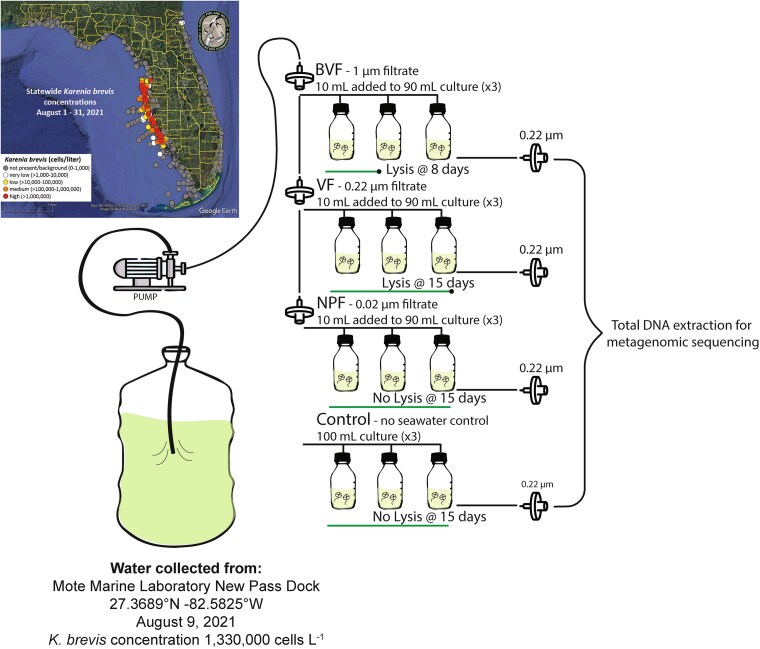
Experimental design for enriching *K. brevis-*specific viruses. Seawater was collected from the Mote Marine Laboratory New Pass dock on 9 August 2021 during a *K. brevis* bloom. Bloom water was sequentially filtered through a 1 μM (BVF), 0.22 μM (VF), and 0.02 μM (NPF) filter. Cultures of *K. brevis* strain CCMP2228 were inoculated with one of the three filtrates and monitored for changes in growth compared to uninoculated controls. Once cell lysis was observed in the BVF cultures (Day 8) and the VF cultures (Day 15), 50 ml samples from each flask were filtered onto a 0.22 μM filter for genomic DNA preservation and subsequent metagenomic sequencing. Control and NPF cultures were processed in the same way on Day 15.

On 9 August 2021, 250 ml of seawater was collected from the Mote Marine Laboratory New Pass Dock, Sarasota, FL (26.71013333, −82.33451667, Supplemental Fig. 1) during a high concentration (1 330 000 cells l^−1^) *K. brevis* bloom. The bloom water was passed through a 100 μM nylon mesh and then sequentially filtered through a 1 μM polycarbonate filter, a 0.22 μM polyethersulfone (PES) filter, and a 0.02 μM Anopore membrane filter to remove different microbial community size classes. At each filtration step, 50 ml aliquots were kept, representing a bacteria and virus enriched filtrate (BVF, <1 μM), a virus enriched filtrate (VF, <0.22 μM), and a nearly particle-free seawater control (NPF, <0.02 μM). The filtrates were shipped overnight to Horn Point Laboratory, Cambridge, MD, where they were separately added at a 10% v/v ratio to triplicate cultures of *K. brevis* (strain CCMP2228, Manasota Key) at a concentration of 10 000 000 cells L^−1^. Triplicate *K. brevis* cultures with no bloom filtrate added were used as negative controls (Fig. 1). Cultures were not axenic, but aseptic techniques were used to limit contamination with microorganisms from any source other than bloom water filtrates.

The *K. brevis* cultures’ maximum photochemical efficiency of photosystem II (*F_v_/F_m_*) was measured on Days 0, 1, 2, 7, 8, 10, 14, and 15 using a Phyto-PAM-II (Heinz Walz GmbH, Effeltrich, Germany) and cell counts of samples preserved with Lugol’s solution were done on Day 0 (before aliquoting the stock culture), Day 8 (for BVF flasks), and Days 14 and 15 (for control, VF, and NPF flasks) as previously described [28, 29]. Bacteria and virus-like population (VLP) abundances were measured on glutaraldehyde-preserved samples using a Becton Dickinson FACScan flow cytometer equipped with a full blue light 488-nm laser, as previously described [30, 31].

The incubation experiment ended when at least two of the BVF- and VF-inoculated *K. brevis* culture replicates lost their color, corresponding to *K. brevis* F*_v_*/F*_m_* and cell count decline (Days 8 and 15, respectively). At these times, 50 ml-aliquots were removed from each flask, filtered through a 0.22 μM PES filter, flash frozen, and stored at −80°C for subsequent DNA extraction and metagenomic sequencing (Fig. 1). Control culture replicate A, BVF replicates A and B, and VF replicates B and C were sent for metagenomic sequencing. To facilitate results comparison, these metagenomes were sequenced at the same read depth (~51.8–55.5 million reads per sample; Supplemental Data 1). The remaining lysed material was left unfiltered and frozen at −80°C for subsequent flow cytometry and viral isolation.

### Identification, annotation, and taxonomy of giant virus metagenome-assembled genomes from the isolation experiment

The metagenomic sequences were assembled with metaSPADES [32] and binned with MetaBAT2 [33]. Giant virus metagenome-assembled genome (GVMAG) completeness and quality were determined using GVClass [34] and CheckV [35]. GVMAG similarity was established based on their average nucleotide identity (ANI) and used to determine viral operational taxonomic units (vOTUs). Gene prediction was done using pyrodigal-gv, and gene annotations were done using geNomad [36].

A GVMAG phylogenomic tree including the GVMAGs from this study and the giant virus reference genomes database (downloaded in September 2024) [37] was generated from the alignment of seven concatenated giant virus default marker genes: Poxvirus Late Transcription Factor VLTF3, A32 Packaging ATPase, DNA topoisomerase II, Transcription initiation factor IIB, DNA polymerase family B, DNA-directed RNA polymerase alpha subunit, and DEAD/SNF2-like helicase [38].

### Analysis to track *Karenia brevis* giant virus and beneficial and algicidal bacteria genomes in coastal Florida waters

The GVMAGs from the isolation experiment were used to recruit reads from nine metagenomes (~32.6–56.6 million reads per metagenome) representing a monthly time series between December 2020 and August 2021, spanning bloom and nonbloom conditions, of coastal Florida seawater samples from station EH25 (offshore Cayo Costa, FL, USA; Supplemental Fig. 1). Metagenome statistics and NCBI accession numbers are provided in *Fei* et al. [17] and Supplemental Data 1. The relative abundance and coverage of environmental metagenomic reads recruited to each GVMAG was calculated using coverm. The GVMAGs relative abundances and *K. brevis* cell counts from EH25 were graphed in R using ggplot2.

Metagenome assemble genomes (MAGs) from the EH25 metagenomes were binned, taxonomically assigned, and their relative abundance estimated as described in [17]. The relative abundance was calculated for bacterial MAGs in the orders *Enterobacterales, Flavobacteriales*, and *Pseudomonadales*, which contain *K. brevis* algicidal members [17], and MAGs identified as *Mameliella alba*, a *K. brevis* beneficial bacteria [17].

### Identification of *Aquintoviricetes* (polinton-like viruses)

The ICTV_VirophageSG tool was used to search for PLVs within the incubation experiment metagenomes by identifying PC_054, a PLV- conserved pATPase [39, 40]. Subsequently, open reading frames (ORFs), predicted using pyrodigal-gv [36], were interrogated using major capsid protein (MCP) Hidden Markov Models [36]. Contigs that contained a PLV MCP with a bit score ≥50 were used to build a PLV MCP phylogenetic tree. All ORFs on contigs that contained both PLV MCP and pATPase were annotated using HHpred [41]. The same PLV search was also applied to the EH25 metagenomes.

See Supplemental Materials and Methods for full details.

## Results and discussion

### *Karenia brevis* culture demise by environmental lytic agents

**Figure 2:**
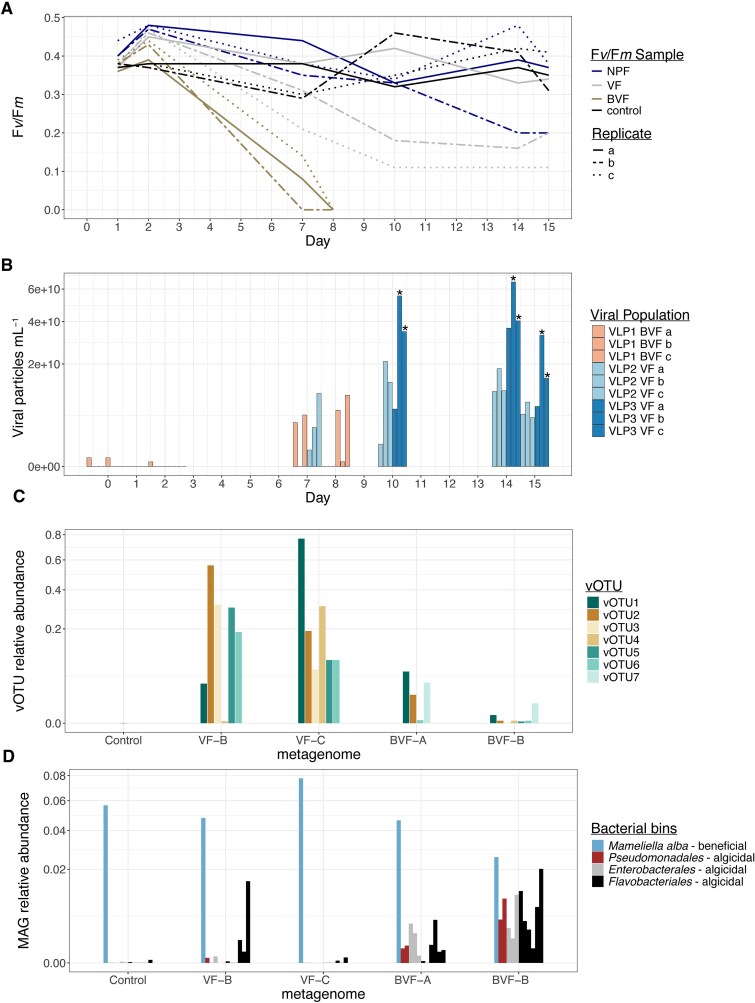
Photophysiological dynamics and virus and bacteria production in *K. brevis* unamended cultures and cultures incubated with 1 μM (BVF), 0.22 μM (VF), and 0.02 μM (NPF) filtered bloom water. (A) Maximum photochemical efficiency of photosystem II, measured as *F_v_/F_m_* over time for each replicate within each treatment: control (black lines), NPF (blue lines), VF (gray lines), and BVF (brown lines). (B) Virus-like particle abundances over time as measured by flow cytometry in the BVF treatment (VLP1, peach bars) and the VF treatment (VLP2, light blue bars; VLP3, dark blue bars). *Karenia brevis F_v_/F_m_* did not decline in the VF replicate A yet VLP populations increased through the incubation. Letters “A, B, C” denote biological replicates within each treatment. * represents statistically significant VLP concentrations when compared to the controls. Corresponding *P*-values are listed in Supplemental Table 3. (C) vOTU relative abundance, based on read mapping, within each metagenome from the incubation experiment. (D) Beneficial and algicidal bacteria MAG relative abundances within each metagenome from the incubation experiment. Each bar represents a bacterial MAG. Blue bars correspond to the beneficial bacteria species *M. alba*; red, gray, and black bars correspond to different MAGs within the algicidal taxa *Pseudomonadales, Enterobacterales*, and *Flavobacteriales*, respectively.

Incubating *K. brevis* strain CCMP2228 with the BVF seawater caused the loss of visible color in all three *K. brevis* culture replicates within eight days. No intact cells were found in those flasks using microscopy. Two out of three *K. brevis* culture replicates incubated with VF seawater were visibly clear by Day 15. Culture color changes corresponded with declines in the maximum photochemical efficiency (F*_v_*/F*_m_*) from ~0.38 ± 0.02 on Day 1, to zero in the BVF incubations (*P* = .001), and ~ 0.15 ± 0.04 in the two cleared VF incubations (replicate B and C) from Day 10 onward (*P* = .013) (Fig. 2A, Supplemental Table 1). The reduction in F*_v_*/F*_m_* may be indicative of the loss of photosynthesis, which is consistent with the loss of cell integrity [42] and the observed loss of color in the culture. F*_v_*/F*_m_* remained relatively constant ~0.39 ± 0.05 in the VF replicate A flask, in replicates A and C incubated with <0.02 μm (NPF) filtered *K. brevis*–bloom seawater (*P* = .7), and in the triplicate unamended culture controls (Fig. 2A). *Karenia brevis* cell counts were between 17 063 and 33 141 cells ml^−1^ in the control flasks on the last day of the experiment, a positive growth rate of 0.067 ± 0.03 divisions day^−1^ from the initial 10 000 cells ml^−1^. Negative growth rates of −0.22 ± 0.04 and −0.06 ± 0.06 divisions day^−1^ with cell counts dropping to ~300–600 cells ml^−1^ and ~2000–4000 cells ml^−1^ occurred in the VF and NPF flasks that lost color, respectively (Supplemental Data 2). *Karenia brevis* growth was negligible by the end of the experiment in the VF (0.01 divisions day^−1^) and NPF (0.003 divisions day^−1^) flasks that maintained color and near constant *F_v_/F_m_* (Fig. 2A, Supplemental Data 2).

Flow cytometry analysis revealed the appearance of giant VLPs (relatively high-side scatter and green fluorescence signatures) in the BVF and VF incubations (Fig. 2B). The abundance of the VLPs was significantly higher in the VF replicates B and C compared to the control incubations (*P* < .01) (Supplemental Table 2). The VLPs abundance was lower in VF replicate A and the difference was not statistically significant compared to the control cultures (*P* = .15) (Fig. 2B, Supplemental Table 2). Notably, the giant VLPs from the BVF and the VF incubations had different fluorescent and side scatter signatures (Supplemental Fig. 2A–C), suggesting filtration through 1 μm-pore size or 0.22 μm-pore size filters led to the propagation of different lytic agents (bacterial or viral) originally present in the bloom seawater samples. The concentration of giant VLPs was significantly higher in the VF than in the BVF incubations (*P* < .05) (Fig. 2B; Supplemental Table 2, Supplemental Data 3). Giant VLPs were not observed in any of the unamended control cultures or the NPF incubations (Supplemental Fig. 2). However, a low-side scatter and low-green fluorescence VLP appeared in the NPF flasks by the end of the experiment (Supplemental Fig. 2D).

### Metagenomic identification and characterization of giant viruses in *Karenia brevis* lysates

Metagenomic sequencing at the same read depth yielded similar (±3%) numbers of raw reads, contigs, and binned genomes from each of the selected control, BVF, and VF replicates (Supplemental Data 1). We recovered a total of 11 *Nucleocytoviricota* GVMAGs between ~408 Kb and ~1.25 Mb in length, with estimated completeness ranging from ~66 to 98.9% (Supplemental Data 4). The shortest and least complete GVMAG originated from the BVF replicate B lysate (BVF_B.bin15), and the other 10 GVMAGs originated from the VF metagenomes. Mapping the reads from the four lysate metagenomes against the GVMAGs showed that BVF_B.bin15 was only present in the BVF flasks (Fig. 2C), suggesting that the corresponding viral particles were either retained by the 0.2 μm-pore size filters used for the VF treatments or that they were infectious to a non-*K. brevis* host introduced in the flask with the <1 μm bloom water filtrate. The GVMAGs from the VF flasks were present in both sequenced VF replicates and in at least one of the BVF metagenomes, albeit at a lower or background relative abundance (~0.1–0.8 in VF metagenomes and <0.1 in the BVF metagenomes) (Fig. 2C). No GVMAGs were found in the control *K. brevis* metagenome (Fig. 2C). These genomic analyses support flow cytometric detection of different VLPs in the BVF and VF lysates but not in the control cultures.

It is unclear why the VF GVMAGs were absent or at very low abundance in the BVF incubations. Physical removal of corresponding viral particles by filtration through 1 μm pore-sized filters (BVF treatment) was not likely, as they were present and propagated in the incubations that received <0.22 μm seawater filtrates (VF treatment). On the contrary, most bacteria would have been removed by filtration from the VF inocula. One possibility is that *K. brevis* lysis in the BVF incubations was primarily caused by algicidal bacteria [18, 19]. Indeed, in an analogous experiment with water collected in March 2021 during earlier stages of the same *K. brevis* bloom, we detected, isolated, and confirmed activities for strains of algicidal *Flavobacteriales* and *Enterobacterales* bacteria from lysed cultures of another *K. brevis* strain (CCMP2229) incubated with <1 μm-filtered bloom water [17]. Additionally, *Pseudomonadales* were metagenomically detected in *K. brevis* lysed incubations, and members in this order are commonly associated with algicidal activity [43]. In tandem, the beneficial (through the provision of vitamins to *K. brevis*) *M. alba* bacterium was isolated from the inoculated and unamended control cultures [17]. In that previous experiment, we showed that the bacterial diversity in the control cultures was stable between the first and last days of the experiment [17]. To assess if bacteria may have prevented GVMAG replication in the BVF incubations, in this study, we calculated the relative abundance of *M. alba, Enterobacterales, Flavobacterales, and Pseudomonadales* in the control, BVF, and VF metagenomes*. Mameliella alba* was present in all the treatments but was more abundant in the VF incubations and control metagenomes (Fig. 2D). The algicidal bacteria were at low abundance, in the control and VF replicate B and C metagenomes (average relative abundances of 2.4^−6^ and 7.7^−4^, respectively), while they were better represented in the BVF metagenomes (average relative abundance of 3.9^−3^) (Fig. 2D). Notably, fewer algicidal bacteria were detected in VF replicate C, from which we assembled six GVMAGs, compared to VF replicate B, from which only four GVMAGs originated. Metagenomic reads from the BVF incubations were recruited to each of the algicidal bacteria taxa (Fig. 2D, Supplemental Data 5). Our results suggest that the relatively fast mortality caused by bacteria in the BVF incubations, eight days for culture clearance versus 15 days in VF flasks, might have hindered the propagation of giant viruses infectious to *K. brevis*. Beneficial bacteria in the VF flasks did not hinder viral lytic activity, possibly because beneficial bacteria aid viral propagation by supporting physiologically active *K. brevis*.

The 11 GVMAGs represented seven lineages of vOTUs, according to MIUViG standards that establish vOTUs in a lineage share at least 95% ANI with an 85% minimum alignment fraction [44]. GVMAGs in vOTUs 2, 3, 5, and 6 were present in both VF metagenomes, vOTUs 1 and 4 were represented by a single GVMAG each (VF_C.bin20 and VF_C.bin27, respectively) from the VF replicate C metagenome, and vOTU7 was represented by the BVF replicate B GVMAG (Supplemental Fig. 3, Supplemental Data 6). Each of the vOTUs’ suite of genes was unique. The conserved functional capabilities included genes involved in maintaining viral DNA integrity, use of host translation machinery, and modulation of host protein expression (Supplemental Fig. 4, Supplemental Data 7).

**Figure 3:**
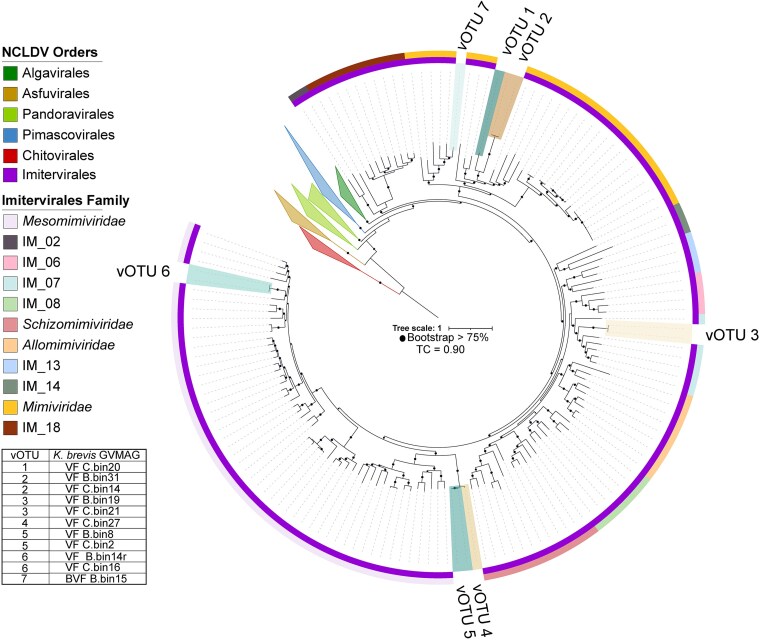
Mid-point rooted *Nucleocytoviricota* conserved marker gene phylogenomic tree showing vOTU1–7 taxonomic affiliations. Bootstrap values were calculated based on 1000 trees, and branch support >75% is represented by black circles. The tree certainty (TC) value is 90%. All GVMAGs identified in this study fall within the order *Imitervirales* (inner dark purple ring). Collapsed clades represent other *Nucleocytoviricota* orders. Outer ring colors denote families within the order *Imitervirales*. Branches with GVMAGs representing each vOTU (listed in bottom left table) are shaded with different colors.

Phylogenomic analyses identified the giant virus vOTUs as novel and placed them within three families in the order *Imitervirales* [37]: *Mimiviridae* (vOTU1), IM-07 (vOTU3), and *Mesomimiviridae* (vOTU4, vOTU5, vOTU6) (Fig. 3). The vOTU1 and vOTU2 GVMAGs were affiliated with the subfamily *Klosneuvirinae* [45] containing Edafosvirus sp. (unknown host, from a soil metagenome), a *Bodo saltans* (flagellated kinetoplastid) virus, and several amoeba (flagellated and nonflagellated) viruses. Notably, Edafosvirus sp. [46] genes had increased transcript expression during the collapse of a *Karenia longicanalis* bloom in Tongxin Bay of Fujian, China [47]. The vOTU3 GVMAGs clustered within the IM_07 family, which includes viruses believed to use the flagellate *Katablepharidaceae* (Alveolata) as a host [48]. The vOTU4 and vOTU5 grouped with *Mesomimiviridae* GVMAGs from freshwater lakes with undetermined hosts [49]. Last, vOTU6 GVMAGs are closely associated with viruses that impact the harmful flagellated bloom-forming phytoplankton species *Phaeocystis globosa* and *Chrysochromulina* sp. [50–53] (Supplemental Fig. 5, Supplemental Data 8).

Noteworthy vOTU genes included dinoflagellate viral nucleoprotein (DVNP, in vOTU6), bacteriorhodopsin (in vOTUs 3–6), and carbonic anhydrase (in vOTU6) (Supplemental Data 7). Dinoflagellate viral nucleoproteins, used as histone replacements in dinoflagellates, are believed to have been acquired from giant viruses [54]. The giant virus DVNP homolog appears to act as a viral-DNA binding protein [55], which aids in condensing their large genomes into the capsids [56]. Copies of the DVNP gene in vOTU6 support a shared genetic history with dinoflagellates. Bacteriorhodopsin is a proton pump regularly found in giant viruses and believed to maintain the host’s proton gradient, thus supporting the host’s ATP synthase [57]. This would enable giant viruses to maintain host energy pools that are needed during viral replication within the cell [58]. Carbonic anhydrase was only identified in vOTU6. The only other account of carbonic anhydrase genes in a giant virus genome that we could find was from a putative fungal virus single amplified genome from a marine igneous crust water sample [59]. Carbonic anhydrase is used by photosynthetic organisms, including *K. brevis*, as a carbon concentrating mechanism, converting bicarbonate to CO_2_ so Rubisco can make organic carbon that aids in cell growth [60]. Additionally, carbonic anhydrase has been implicated in aiding viral infection in virus–plant–insect vector systems. When insect carbonic anhydrase is transmitted to the plant during feeding, the pH of the plant in the wounded area decreases, stimulating plant vesicle circulation, which enhances viral movement and propagation within the plant cells [61]. While currently speculative, it is possible that vOTU6-induced production of carbonic anhydrase aids in carbon availability during viral replication and causes changes in intracellular pH that modulate virion release. The auxiliary bacteriorhodopsin and carbonic anhydrase genes may give vOTU6 viruses a competitive advantage by allowing the virus to maintain energy and carbon availability during viral replication. This could counteract eukaryote defense mechanisms that involve decreasing intracellular carbon and energy pools [62]. Future transcriptomic and physiological studies may help test these ideas.

### Giant viral operational taxonomic units are infectious to *Karenia brevis*

Since *Nucleocytoviricota* infect a wide range of eukaryotes [57], we searched the metagenomes for 18S rRNA gene signatures to check if our incubations contained putative giant virus hosts other than *K. brevis* (see Supplemental Methods). We found no significant evidence of eukaryotic contaminants within the VF metagenomes, where ten GVMAGs were identified. However, the metagenome of the BVF replicate B lysate, from which we recovered the vOTU7 GVMAG, contained 18S rRNA gene sequences that were related to the small phagotrophic flagellate Opalozoa (2–10 μm) [63] (Supplemental Data 9 and 10). While the Opalozoa 18S rRNA gene was not recovered in the BVF replicate A metagenome (by assembly or read mapping Supplemental Data 10), read mapping showed that vOTU7 was also present at low abundance in BVF replicate A (Fig. 2C). Opalozoa 18S rRNA gene sequences were also found intermittently in our environmental Florida Shelf metagenomes, primarily around May, August, and September 2021 (Supplemental Data 10). It is possible these small flagellates were not fully retained by the 1 μM filter and thus were introduced to the BVF incubation flasks. Consequently, we could not unequivocally link the vOTU7 GVMAG from the BVF lysate to *K. brevis* as its host. This GVMAG was, conservatively, removed from downstream analyses. Subsequent 18S rRNA gene amplicon sequencing of the *K. brevis* strain CCMP2228 stock culture confirmed *K. brevis* was the only eukaryote present (Supplemental Data 11), further supporting that Opalozoa were likely introduced with the filtered seawater and that the only possible host for the VF GVMAGs was *K. brevis*.

Phytoplankton species, even at the strain level, can be hosts to a suite of giant virus strains within the same viral species, e.g. *Emiliania huxleyi* [64, 65], *Micromonas pusilla* [66], *P. globosa* [50]. However, there is limited data on single phytoplankton species, or strains, harboring diverse *Nucleocytoviricota* lineages, as found in this study. Two recent studies during independent blooms of the dinoflagellates *P. shikokuense* [24] and *K. longicanalis* [47] in the East China Sea used correlational gene expression analyses to conclude these dinoflagellates were infected by mimiviruses (order *Imitervirales*) and phycodnaviruses (order *Algalvirales*), as well as picorna-like (single-stranded RNA) viruses [47]. Similarly, single-cell isolation and genome-resolved metagenomics showed diverse giant virus associations within amoeba and ciliate hosts and single-cell transcriptomics confirmed gene expression for viruses of the *Mesomimiviridae* family in both host types [67]. It was suggested that other giant virus types associated with the individual amoeba and ciliate cells might infect symbiotic green algae or be ingested as food [67]. While *K. brevis* is a mixotroph [15], the increase in abundance of giant viruses revealed by flow cytometry and read mapping and the lack of evidence of contaminant eukaryotes in our VF incubations support that our vOTU1-vOTU6 GVMAGs were produced through lytic infection of *K. brevis*.

To further confirm a direct connection between vOTU1–6 and *K. brevis*, we inoculated aliquots of two additional *K. brevis* strains (CCMP2820 and CCMP2281) with the VF lysates that had been stored at −80°C for ~10 months (see Supplemental Methods). The inoculated cultures (also free of other eukaryotic contaminants, Supplemental Data 11) cleared after 15 days, and flow cytometry analysis showed the appearance of giant VLPs in the cultures that were incubated with the VF lysates, but not in the noninoculated controls (Supplemental Fig. 6). The VLPs had slightly different fluorescence and side scatter signatures compared to the original lysates (Supplemental Fig. 2), possibly due to the use of a different flow cytometer. These new lysates were used to re-inoculate (in triplicate) the same *K. brevis* strains, resulting in *K. brevis* fluorescence declines by Day 8 (strain CCMP2281 and CCMP2820) (Supplemental Fig. 7A and B). Although one control CCMP2820 replicate culture died, the fluorescence difference was apparent between the other control replicates and the inoculated flasks (Supplemental Fig. 7B). Longer monitoring of the *K. brevis* CCMP2281 showed near complete lack of fluorescence by Day 11 (Supplemental Fig. 7A). The resulting lysates from the two *K. brevis* strains were pooled and screened by PCR with primers designed for the MCP gene of each of the six VF vOTUs (Supplemental Data 12). Only primers designed for vOTU4 and vOTU6 yielded amplicons from the repropagation lysates. Electrophoresis gel analysis revealed several bands in addition to the expected amplicon sizes for these primer sets (Supplemental Fig. 7C). The high similarity across the vOTUs’ MCP genes made designing primers specific to each vOTU a challenge. To confirm the identity of the amplicons, the PCRs with vOTU4 and vOTU6 primers were repeated, and the resulting products were sequenced using Oxford Nanopore. Both primer sets amplified both vOTU5 (primary amplicon from vOTU4 primers) and vOTU6 (primary amplicon from vOTU6 primers) (Supplemental Data 13). The preferential amplification of vOTU5 with the vOTU4 primers is likely due to the high ANI (Supplemental Fig. 3) and close phylogeny (Supplemental Fig. 5) between these two vOTUs. It is possible that the rest of the vOTUs had degraded, lost infectivity during the long-term storage, were not specific to the two new *K. brevis* strains, or their propagation was hindered by other factors, such as hyperparasitism—co-infection with dependent viruses—discussed below.

**Figure 4:**
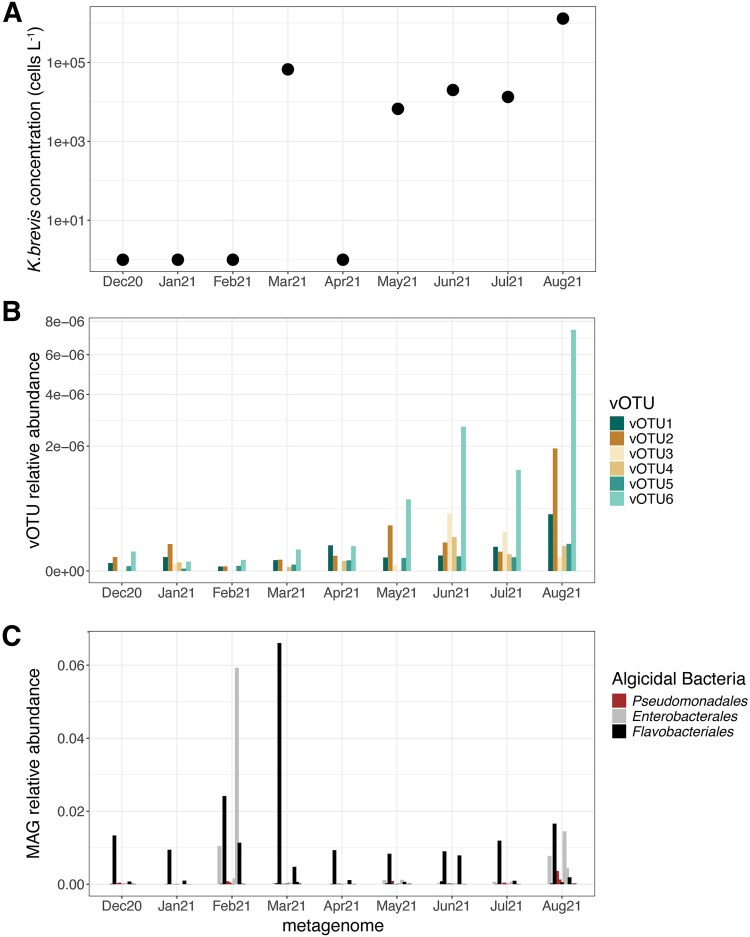
*Karenia brevis* abundance (A) and relative abundances of giant virus vOTUs (B) and algicidal bacteria (C) between December 2020 and August 2021 at station EH25.

Read mapping to environmental metagenomes collected monthly at station EH25 (Supplemental Data 1) detected vOTU1, vOTU2, vOTU5, and vOTU6, and their abundance dynamics paralleled *K. brevis* cell concentrations (*P* = .02–.005) (Fig. 4A and B, Supplemental Fig. 8, Supplemental Data 14). Notably, the vOTU5 and vOTU6 lineages (*Mesomimiviridae*) positively correlated with *K. brevis* concentrations (*P* = .005, *R*^2^ = 0.70) at EH25 during the bloom period (Fig. 4A and B, Supplemental Fig. 8). Both vOTU5 and vOTU6 contained the auxiliary bacteriorhodopsin gene. Additionally, vOTU6 also contained the auxiliary DVNP and carbonic anhydrase genes. The prevalence of vOTU5 and vOTU6 in culture infections and during natural blooms suggests these lineages have a competitive advantage, possibly aided by those auxiliary genes.

Previous studies have inferred viral infection of *K. brevis* in the environment from correlative analysis of total unspecific virus-like particles [26] and metagenome-found ssRNA viral read abundances [27] to *K. brevis* cell abundance. However, to our knowledge, this is the first direct evidence of *K. brevis* lytic viral infection in a laboratory experiment*.* While our study reveals a connection between giant viruses and *K. brevis*, we cannot rule out the presence in the environment of other *K. brevis*-virus types not detected with the methods we employed. Latent (viral genome integration in host genome) and chronic (viral progeny is produced without lysing the host cell) infections can be difficult to detect due to the lack of symptoms typically associated with the presence of viruses, e.g. population crash, reduced growth, or altered metabolism. Additionally, our incubations might not have been long enough to achieve culture clearance by some virus types, as suggested by the results from VF replicate A and the NFP incubations. Also, our metagenomic approach was specific for dsDNA because of the nucleic acid purification and sequencing library preparation methods. Finally, flow cytometry detection of virus-like particles was based on staining with SYBR™ Green I, which has a higher binding affinity for dsDNA. Without further molecular analyses, it is not possible to identify the low-fluorescence, low-side scatter VLP discriminated by flow cytometry in the NPF incubations. Possibilities include phages lytic to beneficial bacteria (e.g. *M. alba*) that indirectly negatively impact *K. brevis* cells as previously suggested [25] or even ssRNA viruses [27].

**Figure 5:**
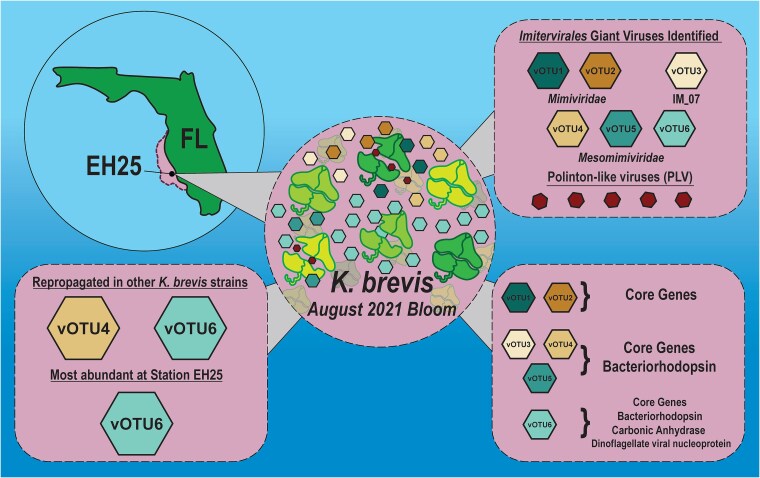
Predicted *K. brevis*–virus–virus interactions during a 2021 bloom along the West Florida Shelf based on findings from this study.

### *Karenia brevis* blooms may persist for extended periods despite active viral infections

*Karenia brevis* is susceptible to infection by diverse dsDNA giant viruses (this study) and, possibly, ssRNA viruses [27]. However, in the environment, the abundance of both of those types of viruses positively correlated with *K. brevis* cell counts during long-lasting blooms, unlike other phytoplankton blooms where the bloom demise coincides with increasing abundances of specific viruses [68–70]. In this study, we show that a dense *K. brevis* bloom in the West Florida Shelf persisted alongside giant viruses (Fig. 4) able to lyse xenic *K. brevis* cultures within 1–2 weeks (Fig. 2). It is increasingly evident that viruses differ in their infection mode, likely occurring within a continuum from lysis to latency spanning antagonistic and beneficial interactions [71, 72]. The mode of infection is attributed to genetic switches [71, 73], the host’s population density [74], and influences from abiotic factors on the host’s physiology [75–79]. Recent environmental studies of the dinoflagellates *P. shikokuense* [24] and *K. longicanalis* [47] suggested that viral infections differently influenced bloom dynamics. Viral infection of *P. shikokuense* appeared to be chronic and did not significantly, contribute to bloom termination [24], while viral infection, in concert with grazing by ciliates and algicidal bacteria, might have led to the collapse of the *K. longicanalis* bloom [47]. Here we have shown that competition with algicidal bacteria can reduce the efficiency and propagation of lytic giant virus infections, possibly by reducing the availability of suitable hosts and, consequently, of host-virus encounters.

We also retrieved three PLVs (*Aquintoviricetes* [39]) partial genomes from our lysate metagenomes: PLV1-VF_B (6133 bp), PLV2-VF_C (2492 bp), and PLV3-BVF_ B (10 425 bp) (Supplemental Data 15, Supplemental Fig. 9). PLVs are small eukaryotic viruses (~60–80 nm, 15–40 Kb) that resemble transposable elements and can integrate into algal genomes [39, 80]. Many PLVs are believed to be hyperparasitic, i.e. their propagation depends on co-infection with a helper giant virus, as they require the giant virus transcription machinery [39]. Tandem replication of PLV and giant viruses within the same host cell decreases giant virus replication efficiency and burst size, acting as a constraint on giant virus lytic activity and propagation [81]. PLVs have been found integrated into *Symbiodinium* dinoflagellates and are predicted to compose 1%–2% of dinoflagellate genomes [80].

The PLV1 and PLV2 contigs were found in the metagenome from the VF (<0.22 μM) replicates B and C incubation at 92× and 2× coverage, respectively. PLV3 was recovered from the BVF (<1 μM) replicate B metagenome at a 4× coverage (Supplemental Data 15). Despite not being complete genomes, the relatively high read coverage of these contigs suggests active PLV replication [82]. The higher coverage and relative abundance of PLV1 coincides with the higher abundance of giant viruses in the VF replicate B metagenome (*P* < .001) (Supplemental Fig. 10, Supplemental Table 3). PLVs were also detected in the environment (at station EH25) in September 2021 (Supplemental Data 16), following the August 2021 peak in giant virus vOTU abundance. The lack of detection of PLVs in the control culture metagenome does not necessarily indicate their absence as integrated viruses in *K. brevis*, instead it may reflect inefficient *K. brevis* cell lysis and DNA purification during sample preparation or insufficient sequencing depth. While admittedly speculative at this time, *K. brevis*-associated PLVs may act as a defense mechanism, reducing the virulent impact of giant viruses in subsequent propagations. It appears that, under our experimental conditions, giant virus infection might have been only partially limited by PLVs, as suggested by the lack of complete culture clearance by the end of the incubation (Fig. 2, Supplemental Data 2). Consequently, it seems reasonable to suggest that PLV interference combined with optimal *K. brevis* growth conditions [14] may contribute to bloom maintenance and help explain a positive correlation between *K. brevis* and specific giant virus abundance. Conversely, giant viruses may be more successful at lysing *K. brevis* cells in concert with other stressors, e.g. suboptimal temperature, as in our experiment, where cultures were incubated at 20°C. *Karenia brevis* has been shown to survive at temperatures between 7°C and 33°C in the field [7], although the reported optimal temperature range is 22°C–28°C [14].

## Implications for understanding *Karenia brevis* bloom ecology

This study describes giant viruses and possible PLVs that propagate using *K. brevis,* unveiling another layer of complexity in factors influencing bloom dynamics. This adds to accumulating evidence that *K. brevis* blooms harbor diverse microbiomes, and in conjunction with physicochemical factors, direct and indirect interactions between *K. brevis* and its associated microbes may influence the initiation and longevity of blooms. We hypothesize a scenario in which the environmental conditions (e.g. ocean circulation, nutrient supply, and temperature) determine the balance and direction of the interactions among microbiome members with each other and with *K. brevis*. For example, we previously showed that enriching *K. brevis* plus beneficial bacteria cocultures with microbes present during the early stages of a natural bloom leads to a quick *K. brevis* population decline driven by certain taxa of algicidal bacteria [17]. In this follow-up study, we further show that the reduction of algicidal bacteria creates an opportunity for giant viruses to infect and lyse *K. brevis* populations (Fig. 2C and D, Fig. 4B and C). In turn, giant virus replication and transcription may support the propagation of PLVs, decreasing the subsequent virulent impact of giant viruses, possibly enhancing population maintenance and bloom persistence in the environment (Fig. 5). Future experimental research and field observations are required to disentangle the precise interactions between specific microbial players and *K. brevis* to better understand the mechanisms and their effect on bloom fate.

Our findings may have direct implications for management strategies, e.g. through the monitoring of specific microbial taxa abundance and dynamics, allowing earlier and more accurate prediction of bloom stages, particularly termination.

## Supplementary Material

ycag051_Supplementary_material

## Data Availability

All shotgun metagenomic reads analyzed in this study are deposited on NCBI with specific identification numbers listed in Supplemental Data 1. All assembled GVMAGs (vOTU1–6) are deposited in GenBank, and accession identifiers are listed in Supplemental Data 4.

## References

[1] Anderson DM, Cembella AD, Hallegraeff GM. Progress in understanding harmful algal blooms: paradigm shifts and new Technologies for research, monitoring, and management. *Ann Rev Mar Sci* 2012;4:143–76. 10.1146/annurev-marine-120308-081121 PMC5373096 22457972

[2] Dai Y, Yang S, Zhao D. et al. Coastal phytoplankton blooms expand and intensify in the 21st century. *Nature* 2023;615:280–4. 10.1038/s41586-023-05760-y PMC9995273 36859547

[3] Glibert PM, Icarus Allen J, Artioli Y. et al. Vulnerability of coastal ecosystems to changes in harmful algal bloom distribution in response to climate change: projections based on model analysis. *Glob Chang Biol* 2014;20:3845–58. 10.1111/gcb.12662 24942916

[4] Townhill BL, Tinker J, Jones M. et al. Harmful algal blooms and climate change: exploring future distribution changes. *ICES J Mar Sci* 2018;75:1882–93. 10.1093/icesjms/fsy113

[5] CA Heil, SA Amin, PM Glibert, KA Hubbard, M Li, J Martínez Martínez. et al. Termination patterns of Karenia brevis blooms in the eastern Gulf of Mexico. In: 19th Int. Conf. Harmful Algae, La Paz, B.C.S., Mex. Soc. Study Harmful Algal Bloom. 2022. 10.5281/zenodo.7034923

[6] Feng L, Wang Y, Hou X. et al. Harmful algal blooms in inland waters. *Nat Rev Earth Environ* 2024;5:631–44. 10.1038/s43017-024-00578-2 PMC11849997 39995947

[7] Brand LE, Campbell L, Bresnan E. Karenia: the biology and ecology of a toxic genus. *Harmful Algae* 2012;14:156–78. 10.1016/j.hal.2011.10.020 36733478 PMC9891709

[8] Newborn S . Red tide is causing fish kills along Pinellas beaches. In: Heal News Florida. USA: WUSF Public Media, 2022.

[9] Flewelling LJ, Naar JP, Abbott JP. et al. Red tides and marine mammal mortalities. *Nature* 2005;435:755–6. 10.1038/nature435755a PMC2659475 15944690

[10] Backer LC, Fleming LE, Rowan A. et al. Recreational exposure to aerosolized brevetoxins during Florida red tide events. *Harmful Algae* 2003;2:19–28. 10.1016/S1568-9883(03)00005-2

[11] Hoagland AP, Anderson DM, Kaoru Y. et al. The economic effects of harmful algal blooms in the United States: estimates, assessment issues, and information needs. *Estuaries* 2002;25:819–37. 10.1007/BF02804908

[12] Fleming LE, Kirkpatrick B, Backer LC. et al. Review of Florida red tide and human health effects. *Harmful Algae* 2011;10:224–33. 10.1016/j.hal.2010.08.006 PMC3014608 21218152

[13] Heil CA, Muni-Morgan AL. Florida’s harmful algal bloom (HAB) problem: escalating risks to human, environmental and economic health with climate change. *Front Ecol Evol* 2021;9:646080. 10.3389/fevo.2021.646080

[14] Vargo GA . A brief summary of the physiology and ecology of Karenia brevis Davis (G. Hansen and Moestrup comb. nov.) red tides on the West Florida shelf and of hypotheses posed for their initiation, growth, maintenance, and termination. *Harmful Algae* 2009;8:573–84. 10.1016/j.hal.2008.11.002

[15] Glibert PM, Burkholder JAM, Kana TM. et al. Grazing by *Karenia brevis* on *Synechococcus* enhances its growth rate and may help to sustain blooms. *Aquat Microb Ecol* 2009;55:17–30. 10.3354/ame01279

[16] Ahn SH, Glibert PM. Temperature-dependent Mixotrophy in natural populations of the toxic dinoflagellate Karenia brevis. *Water* 2024;16:1555. 10.3390/w16111555

[17] Fei C, Booker A, Klass S. et al. Friends and foes: symbiotic and algicidal bacterial influence on Karenia brevis blooms. *ISME Commun* 2025;5:ycae164. 10.1093/ismeco/ycae164 PMC11740886 39830096

[18] Mayali X, Doucette GJ. Microbial community interactions and population dynamics of an algicidal bacterium active against Karenia brevis (Dinophyceae). *Harmful Algae* 2002;1:277–93. 10.1016/S1568-9883(02)00032-X

[19] Roth PB, Twiner MJ, Mikulski CM. et al. Comparative analysis of two algicidal bacteria active against the red tide dinoflagellate Karenia brevis. *Harmful Algae* 2008;7:682–91. 10.1016/j.hal.2008.02.002

[20] Takahashi M, Masuda Y, Chiba Y. et al. dsRNA sequencing revealed a previously missed terminal sequence of a +ssRNA virus that infects dinoflagellate Heterocapsa circularisquama. *Virus Genes* 2024;60:97–99. 10.1007/s11262-023-02046-3 38198069

[21] Tomaru Y, Hata N, Masuda T. et al. Ecological dynamics of the bivalve-killing dinoflagellate Heterocapsa circularisquama and its infectious viruses in different locations of western Japan. Environ Microbiol 2007;9:1376–83. 10.1111/j.1462-2920.2007.01252.x 17504475

[22] Nagasaki K, Tomaru Y, Tarutani K. et al. Growth characteristics and Intraspecies host specificity of a large virus infecting the dinoflagellate Heterocapsa circularisquama. *Appl Environ Microbiol* 2003;69:2580–6. 10.1128/AEM.69.5.2580-2586.2003 PMC154496 12732524

[23] Kim JJ, Kim CH, Takano Y. et al. Isolation and physiological characterization of a new Algicidal virus infecting the harmful dinoflagellate Heterocapsa pygmaea. *Plant Pathol J* 2012;28:433–8. 10.5423/PPJ.NT.07.2012.0093

[24] Wang J, Li L, Lin S. Active viral infection during blooms of a dinoflagellate indicates dinoflagellate-viral co-adaptation. *Appl Environ Microbiol* 2023;89:e01156–23. 10.1128/aem.01156-23 PMC10686096 37874280

[25] Paul JH, Houchin L, Griffin D. et al. A filterable lytic agent obtained from a red tide bloom that caused lysis of Karenia brevis (Gymnodinum breve) cultures. *Aquat Microb Ecol* 2002;27:21–27. 10.3354/ame027021

[26] Meyer KA, O’Neil JM, Hitchcock GL. et al. Microbial production along the West Florida shelf: responses of bacteria and viruses to the presence and phase of Karenia brevis blooms. *Harmful Algae* 2014;38:110–8. 10.1016/j.hal.2014.04.015

[27] Lim SJ, Rogers A, Rosario K. et al. Diverse ssRNA viruses associated with Karenia brevis harmful algal blooms in Southwest Florida. *mSphere* 2025;10:e01090–224. 10.1128/msphere.01090-24 PMC12039238 40111022

[28] Ahn, Sophia SH, Glibert PM. Shining light on photosynthesis in the harmful dinoflagellate Karenia mikimotoi–responses to short-term changes in temperature, nitrogen form, and availability. *Phycology.* 2021;2:30–44. 10.3390/phycology2010002

[29] Murchie EH, Lawson T. Chlorophyll fluorescence analysis: a guide to good practice and understanding some new applications. *J Exp Bot* 2013;64:3983–98. 10.1093/jxb/ert208 23913954

[30] Marie D, Partensky F, Vaulot D. et al. Enumeration of phytoplankton, bacteria, and viruses in marine samples. *Curr Protoc Cytom* 1999;10:11.11.1-11.11.15. 10.1002/0471142956.cy1111s10 18770685

[31] Brussaard CPD . Optimization of procedures for counting viruses by flow cytometry. *Appl Environ Microbiol* 2004;70:1506–13. 10.1128/AEM.70.3.1506-1513.2004 PMC368280 15006772

[32] Nurk S, Meleshko D, Korobeynikov A. et al. metaSPAdes: a new versatile metagenomic assembler. *Genome Res* 2017;27:824–34. 10.1101/gr.213959.116 PMC5411777 28298430

[33] Kang DD, Froula J, Egan R. et al. MetaBAT, an efficient tool for accurately reconstructing single genomes from complex microbial communities. *PeerJ* 2015;3:e1165. 10.7717/peerj.1165 26336640 PMC4556158

[34] Pitot TM, Brůna T, Schulz F. Conservative taxonomy and quality assessment of giant virus genomes with GVClass. *npj Viruses* 2024;2:60. 10.1038/s44298-024-00069-7 PMC11721457 40295812

[35] Nayfach S, Camargo AP, Schulz F. et al. CheckV assesses the quality and completeness of metagenome-assembled viral genomes. *Nat Biotechnol* 2021;39:578–85. 10.1038/s41587-020-00774-7 PMC8116208 33349699

[36] Camargo AP, Roux S, Schulz F. et al. Identification of mobile genetic elements with geNomad. *Nat Biotechnol* 2023;42:1303–12. 10.1038/s41587-023-01953-y PMC11324519 37735266

[37] Aylward FO, Moniruzzaman M, Ha AD. et al. A phylogenomic framework for charting the diversity and evolution of giant viruses. *PLoS Biol* 2021;19:e3001430. 10.1371/journal.pbio.3001430 PMC8575486 34705818

[38] Moniruzzaman M, Martinez-Gutierrez CA, Weinheimer AR. et al. Dynamic genome evolution and complex virocell metabolism of globally-distributed giant viruses. *Nat Commun* 2020;11:1710. 10.1038/s41467-020-15507-2 PMC7136201 32249765

[39] Bellas CM, Sommaruga R. Polinton-like viruses are abundant in aquatic ecosystems. *Microbiome.* 2021;9:13. 10.1186/s40168-020-00956-0 PMC7805220 33436089

[40] Koonin EV, Fischer MG, Kuhn JH. et al. The polinton-like supergroup of viruses: evolution, molecular biology, and taxonomy. *Microbiol Mol Biol Rev* 2024;88:e00086–23. 10.1128/mmbr.00086-23 PMC11426020 39023254

[41] Zimmermann L, Stephens A, Nam SZ. et al. A completely Reimplemented MPI bioinformatics toolkit with a new HHpred server at its Core. *J Mol Biol* 2018;430:2237–43. 10.1016/j.jmb.2017.12.007 29258817

[42] Maxwell K, Johnson GN. Chlorophyll fluorescence—a practical guide. *J Exp Bot* 2000;51:659–68. 10.1093/jexbot/51.345.659 10938857

[43] Hotter V, Zopf D, Kim HJ. et al. A polyyne toxin produced by an antagonistic bacterium blinds and lyses a Chlamydomonad alga. *Proc Natl Acad Sci* 2021;118:e2107695118. 10.1073/pnas.2107695118 PMC8379975 34389682

[44] Roux S, Adriaenssens EM, Dutilh BE. et al. Minimum information about an uncultivated virus genome (MIUViG). *Nat Biotechnol* 2019;37:29–37. 10.1038/nbt.4306 PMC6871006 30556814

[45] Andreani J, Schulz F, Di Pinto F. et al. Morphological and genomic features of the new Klosneuvirinae isolate Fadolivirus IHUMI-VV54. *Front Microbiol* 2021;12:719703. 10.3389/fmicb.2021.719703 PMC8490762 34621250

[46] Schulz F, Alteio L, Goudeau D. et al. Hidden diversity of soil giant viruses. *Nat Commun* 9:4881. 10.1038/s41467-018-07335-2 PMC6243002 30451857

[47] Yu L, Li T, Li H. et al. In situ molecular ecological analyses illuminate distinct factors regulating formation and demise of a harmful dinoflagellate bloom. *Microbiol Spectrum* 2023;11:e05157–22. 10.1128/spectrum.05157-22 PMC10269597 37074171

[48] Fromm A, Hevroni G, Vincent F. et al. Single-cell RNA-seq of the rare virosphere reveals the native hosts of giant viruses in the marine environment. *Nat Microbiol* 2024;9:1619–29. 10.1038/s41564-024-01669-y PMC11265207 38605173

[49] Schulz F, Roux S, Paez-Espino D. et al. Giant virus diversity and host interactions through global metagenomics. *Nature* 2020;578:432–6. 10.1038/s41586-020-1957-x PMC7162819 31968354

[50] Baudoux AC, Brussaard CPD. Characterization of different viruses infecting the marine harmful algal bloom species *Phaeocystis globosa*. *Virology* 2005;341:80–90. 10.1016/j.virol.2005.07.002 16081120

[51] Sandaa R-A, Heldal M, Castberg T. et al. Isolation and characterization of two viruses with large genome size infecting Chrysochromulina ericina (Prymnesiophyceae) and Pyramimonas orientalis (Prasinophyceae). *Virology* 2001;290:272–80. 10.1006/viro.2001.1161 11883191

[52] Mars, Brisbin M, Mitarai S, Saito MA. et al. Microbiomes of bloom-forming Phaeocystis algae are stable and consistently recruited, with both symbiotic and opportunistic modes. *ISME J* 2022;16:2255–64. 10.1038/s41396-022-01263-2 PMC9381791 35764675

[53] Fon M, Šupraha L, Andersen T. et al. Optimal growth conditions of the haptophyte Chrysochromulina leadbeateri causing massive fish mortality in northern Norway. *Harmful Algae* 2024;139:102709. 10.1016/j.hal.2024.102709 39567085

[54] Irwin NAT, Martin BJE, Young BP. et al. Viral proteins as a potential driver of histone depletion in dinoflagellates. *Nat Commun* 2018;9:1535. 10.1038/s41467-018-03993-4 PMC5906630 29670105

[55] Wang H, Meng L, Otaegi-Ugartemendia S. et al. Haptophyte-infecting viruses change the genome condensing proteins of dinoflagellates. *Commun Biol* 2025;8:510. 10.1038/s42003-025-07905-3 PMC11953307 40155463

[56] Talbert PB, Henikoff S, Armache K-J. Giant variations in giant virus genome packaging. *Trends Biochem Sci* 2023;48:1071–82. 10.1016/j.tibs.2023.09.003 37777391

[57] Schulz F, Abergel C, Woyke T. Giant virus biology and diversity in the era of genome-resolved metagenomics. *Nat Rev Microbiol* 2022;20:721–36. 10.1038/s41579-022-00754-5 35902763

[58] Needham DM, Yoshizawa S, Hosaka T. et al. A distinct lineage of giant viruses brings a rhodopsin photosystem to unicellular marine predators. *Proc Natl Acad Sci* 2019;116:20574–83. 10.1073/pnas.1907517116 PMC6789865 31548428

[59] Bhattacharjee AS, Schulz F, Woyke T. et al. Genomics discovery of giant fungal viruses from subsurface oceanic crustal fluids. *ISME Commun* 2023;3:10. 10.1038/s43705-022-00210-8 PMC9894930 36732595

[60] Errera RM, Yvon-Lewis S, Kessler JD. et al. Reponses of the dinoflagellate Karenia brevis to climate change: pCO2 and sea surface temperatures. *Harmful Algae* 2014;37:110–6. 10.1016/j.hal.2014.05.012

[61] Guo H, Zhang Y, Li B. et al. Salivary carbonic anhydrase II in winged aphid morph facilitates plant infection by viruses. *Proc Natl Acad Sci* 2023;120:e2222040120. 10.1073/pnas.2222040120 PMC10083582 36976769

[62] Queiroz VF, Tatara JM, Botelho BB. et al. The consequences of viral infection on protists. *Commun Biol* 2024;7:306. 10.1038/s42003-024-06001-2 PMC10925606 38462656

[63] Cho A, Tikhonenkov DV, Lax G. et al. Phylogenomic position of genetically diverse phagotrophic stramenopile flagellates in the sediment-associated MAST-6 lineage and a potentially halotolerant placididean. *Mol Phylogenet Evol* 2024;190:107964. 10.1016/j.ympev.2023.107964 37951557

[64] Wilson WH, Tarran GA, Schroeder D. et al. Isolation of viruses responsible for the demise of an Emiliania huxleyi bloom in the English Channel. *J Mar Biol Assoc UK* 2002;82:369–77. 10.1017/S002531540200560X

[65] Martínez, Martínez J, Schroeder DC, Wilson WH. Dynamics and genotypic composition of Emiliania huxleyi and their co-occurring viruses during a coccolithophore bloom in the North Sea. *FEMS Microbiol Ecol* 2012;81:315–23. 10.1111/j.1574-6941.2012.01349.x 22404582

[66] Martínez, Martínez J, Boere A, Gilg I. et al. New lipid envelope-containing dsDNA virus isolates infecting Micromonas pusilla reveal a separate phylogenetic group. *Aquat Microb Ecol* 2015;74:17–28. 10.3354/ame01723

[67] Schulz F, Yan Y, Weiner AKM. et al. Protists as mediators of complex microbial and viral associations. *bioRxiv* 2024. 10.1101/2024.12.29.630703

[68] Martínez, Martínez J, Schroeder DC, Larsen A. et al. Molecular dynamics of Emiliania huxleyi and Cooccurring viruses during two separate Mesocosm studies. *Appl Environ Microbiol* 2007;73:2. 10.1128/AEM.00864-06 PMC1796978 17098923

[69] Vieira HH, Bulzu P-A, Kasalický V. et al. Isolation of a widespread giant virus implicated in cryptophyte bloom collapse. *ISME J* 2024;18:wrae029. 10.1093/ismejo/wrae029 PMC10960955 38401169

[70] Biggs TEG, Huisman J, Brussaard CPD. Viral lysis modifies seasonal phytoplankton dynamics and carbon flow in the Southern Ocean. *ISME J* 2021;15:3615–22. 10.1038/s41396-021-01033-6 PMC8630045 34155334

[71] Correa AMS, Howard-Varona C, Coy SR. et al. Revisiting the rules of life for viruses of microorganisms. *Nat Rev Microbiol* 2021;19:501–13. 10.1038/s41579-021-00530-x 33762712

[72] Weitz JS, Li G, Gulbudak H. et al. Viral invasion fitness across a continuum from lysis to latency†. *Virus Evol* 2019;5:vez006. 10.1093/ve/vez006 PMC6476163 31024737

[73] Miller G . The switch between latency and replication of Epstein-Barr virus. *J Infect Dis* 1990;161:833–44. 10.1093/infdis/161.5.833 2157769

[74] Knowles B, Bonachela JA, Behrenfeld MJ. et al. Temperate infection in a virus–host system previously known for virulent dynamics. *Nat Commun* 2020;11:4626. 10.1038/s41467-020-18078-4 PMC7493887 32934228

[75] Howard-Varona C, Lindback MM, Fudyma JD. et al. Environment-specific virocell metabolic reprogramming. *ISME J* 2024;18:wrae055. 10.1093/ismejo/wrae055 PMC11170926 38552150

[76] McParland EL, Wright A, Art K. et al. Evidence for contrasting roles of dimethylsulfoniopropionate production in Emiliania huxleyi and Thalassiosira oceanica. *New Phytol* 2020;226:396–409. 10.1111/nph.16374 31850524 PMC7154784

[77] Vega, Thurber RL, Barott KL, Hall D. et al. Metagenomic analysis indicates that stressors induce production of herpes-like viruses in the coral Porites compressa. *Proc Natl Acad Sci* 2008;105:18413–8. 10.1073/pnas.0808985105 PMC2584576 19017800

[78] Correa AMS, Ainsworth TD, Rosales SM. et al. Viral outbreak in corals associated with an In situ bleaching event: atypical herpes-like viruses and a new Megavirus infecting Symbiodinium. *Front Microbiol* 2016;7:127. 10.3389/fmicb.2016.00127 PMC4761846 26941712

[79] Brum JR, Hurwitz BL, Schofield O. et al. Seasonal time bombs: dominant temperate viruses affect Southern Ocean microbial dynamics. *ISME J* 2016;10:437–49. 10.1038/ismej.2015.125 PMC4737935 26296067

[80] Bellas C, Hackl T, Plakolb M-S. et al. Large-scale invasion of unicellular eukaryotic genomes by integrating DNA viruses. *Proc Natl Acad Sci* 2023;120:e2300465120. 10.1073/pnas.2300465120 PMC10120064 37036967

[81] Roitman S, Rozenberg A, Lavy T. et al. Isolation and infection cycle of a polinton-like virus virophage in an abundant marine alga. *Nat Microbiol* 2023;8:332–46. 10.1038/s41564-022-01305-7 36702941

[82] Barth ZK, Hicklin I, Thézé J. et al. Genomic analysis of hyperparasitic viruses associated with entomopoxviruses. *Virus Evol* 2024;10:veae051. 10.1093/ve/veae051 PMC11296320 39100687

