# Supplementary material for: Complex viral interactions revealed for the harmful bloom-forming dinoflagellate Karenia brevis
Source: ISME Commun. 2026 Mar 9;6(1):ycag051. doi: 10.1093/ismeco/ycag051 (PMC13037468; doi:10.1093/ismeco/ycag051)
Supplement: ycag051_Supplementary_material [file ycag051_supplementary_material.zip › Supplemental_Material_12242025.docx]

**Supplemental Methods**

*Karenia brevis* culture maintenance

Non-axenic *K. brevis* cultures (strains CCMP2228, CCMP2820, and CCMP2281) were maintained in L1 medium [1] minus silica at a salinity of 35 at either 20°C (CCMP2228) or 24°C (CCMP2820 and CCMP2281) under a light intensity of 125 μmol photons m^-2^ s^-1^ on a 12:12 light:dark cycle. The L1 media was prepared with 0.22 μM-filtered autoclaved seawater collected offshore from Sarasota Bay, Florida for CCMP2228, or offshore from the Damariscotta River, Maine for CCMP2820 and CCMP2281.

Temporal sampling to monitor *K. brevis* and virus abundances along the Florida coast

To monitor the presence of marine viruses along the west Florida Shelf during the 2021 *K. brevis* bloom, 2-5 L of seawater was collected from sampling location EH25 off the coast of Cayo Costa, FL, USA from December 2020 – September 2021, and March 2022. All sampled seawater was sequentially filtered through 100 μM mesh, then 10 μM, 3 μM, and 0.22 μM polycarbonate membrane filters. Filters were stored in individual cryotubes, flash frozen in liquid nitrogen, and stored at -80°C until DNA extraction. Only 0.22 μM filters were used for subsequent EH25 metagenomic analyses.

In tandem, to monitor the concentration of *K. brevis* cells at EH25, a 5mL aliquot of unfiltered seawater was preserved with 5% Lugol’s iodine until ready for microscopy enumeration.

Total DNA extraction, metagenomic sequencing, and read processing from *K. brevis* viral isolation experiment and EH25 environmental metagenomes

Fifty milliliter aliquots from each incubation flask were filtered onto 0.22 μM-pore sized PES filters on incubation days 8 (for BVF flasks, amended with <1 μM bloom water filtrates) or 15 (for no bloom water control cultures, VF < 0.22 μM bloom water filtrate, and NPF <0.02 μM bloom water filtrate treatments). Total DNA was extracted from the 0.22 μM filters using DNeasy^®^ PowerWater^®^ Kits (Qiagen, MD, USA) following standard instructions except for 10 minutes of bead beating, and each centrifugation length was 1.5 minutes. The same procedure was used to extract DNA from the eleven EH25 0.22 μM filters.

Due to budget limitations, we selected control culture replicate A, BVF replicates A and B, and VF replicates B and C for metagenomic sequencing. The DNA samples were shipped to the University of Illinois at Urbana-Champaign Roy J. Carver Biotechnology Center DNA Services Lab. Shotgun DNA libraries were prepared with the xGen^TM^ ssDNA-Low Input DNA library prep kit (IDT, IA, USA) with a target size of 150 bp paired-end reads. The libraries were pooled, quantitated by qPCR, and sequenced on one NovaSeq 6000 SP lane (Illumina, CA, USA) for 151 cycles from both fragment ends.

Fastq files were generated from the Illumina raw reads (150 bps PE) and demultiplexed with the bcl2fastq v2.20 Conversion Software (Illumina, CA, USA). Corresponding MultiQC reports found no samples with any adapter contamination >0.1%. Quality filtering was done using the bbduk.sh script from bbmap v35.85 [2] to remove phiX and low quality sequences with options “ref= bbmap/35.85/resources/phix174_ill.ref.fa.gz k=27 hdist=1 qtrim=rl trimq=17 cardinality=t mingc=0.05 maxgc=0.95 -Xmx30g”. Remaining reads were checked for common mammalian contaminants (ex. human, cat, dog, mouse) using bbmap v35.85 [2] and the reference database here (https://portal.nersc.gov/dna/microbial/assembly/bushnell/). Forward and reverse reads were interleaved using the reformat.sh script from bbmap v35.85 [2], subsequently reads were filtered with bfc v181 [3] using options “-l -s 10g -k21” to correct or remove unique kmers [4].

Quality-controlled reads from each metagenome, were assembled within each sample using metaSPADES v3.15.4 [5] with option “-k 21,33,55,77,99,127”. Sequences were submitted to NCBI under BioProject PRJNA1077797 and GVMAGs can be found under GenBank accession numbers PV483752-PV483756, PV637767-PV637835.

The metagenomic contigs were binned based on read-depth using bbmap v35.85 [2], samtools v1.15.1 [6], and MetaBAT v2.15 [7]. Giant virus bins were identified using GVClass v0.9.4 [8]. Giant virus metagenome assembled genome (GVMAG) completeness and quality were determined using GVCLASS and CheckV v0.9.0 [9]. Gene prediction was done using pyrodigal-gv v 3.5.1. Viral gene annotations were done using geNomad v1.7.4 [10] with a bitscore of 50 and higher.

The GVMAGs were compared based on their average nucleotide identity, calculated using ANIclustermap v1.1.0 (https://github.com/moshi4/ANIclustermap). To generate concatenated giant virus phylogenomic trees, ncldv_markersearch with “-c” option was used to identify, extract, concatenate, and align the default seven giant virus marker genes (Poxvirus Late Transcription Factor VLTF3, A32 Packaging ATPase, DNA topoisomerase II, Transcription initiation factor IIB, DNA polymerase family B, DNA-directed RNA polymerase alpha subunit, & DEAD/SNF2-like helicase) [11]. This analysis included the 10 giant virus bins from this study, and the giant virus database reference genomes downloaded September 2024 [12]. The concatenated giant virus genomes were subsequently trimmed using trimAl v1.4.22 [13][82] using “-automated1”. The resulting trimmed alignment file was used as input for IQ-Tree v2.2.2.6 [14] using parameters “-m MFP -bb 1000 -nt AUTO -wbt”. The resulting tree was midpoint rooted, and annotated based on previously published giant virus trees in iTOL [11, 12]. RAxML v8.2.12 was used to calculate tree certainty scores using options “-f i -m GTRCAT” [15].

The GVMAGs from the incubation experiment were used to recruit metagenomic reads from the EH25 station samples. The relative abundance and coverage of environmental metagenomic reads recruited to each GVMAG was calculated using coverm v0.6.1 using options --min-read-percent-identity 90 --min-read-aligned-percent 75 --min-covered-fraction 0 --minimap2-parameters "-I 500M" (<https://github.com/wwood/CoverM?tab=readme-ov-file#coverm>). Coverm relative abundance calculations and *K. brevis* cell counts from EH25 were graphed in R using ggplot2.

Read mapping to determine the abundance of beneficial and algicidal bacteria was done using Salmon and BBsplit as described in [16].

18S rRNA gene amplicon sequencing for *K. brevis* cultures

To validate *K. brevis* was the only eukaryotic host present in cultures used for viral propagation, 30mL of unamended culture from *K. brevis* strain Manasota Key cultures (CCMP2228), strain (CCMP2820), and strain (CCMP2281) were filtered through a 0.22 μM filter. Total DNA was extracted from each filter using extraction methods described above. Total DNA was sent for full-length 18S HiFi PacBio sequencing at the Dalhousie University Integrated Microbiome Resource center. This generated 123961 sequences. These sequences were processed and taxonomically identified using the Bigelow Laboratory for Ocean Sciences eDNA-dada2 pipeline (<https://github.com/BigelowLab/edna-dada2/wiki/Processing-PacBio-reads>). To identify if eukaryotes were added through the BVF or VF filtered bloom water, the metagenomes from the enrichment experiment were passed through CMsearch v1.1.4 [17] using the Rfam (RF01960) eukaryotic small subunit ribosomal RNA as the sequence database.

Repropagation of vOTUs

In Summer 2022, August 2021 BVF and VF lysates were inoculated into duplicate *K. brevis* cultures CCMP2820 and CCMP2281 at a 15% v/v ratio that had a starting concentration of 24,000,000 and 20,000,000 cells/L, respectively. Duplicate *K. brevis* cultures with no bloom filtrate added were used as controls. Both *K. brevis* strains were maintained in L1 medium [1] minus silica at a salinity of 31. Culture incubation conditions were 125 μmol of light on a 12-hour light:12-hour dark cycle at 24°C. Seawater used in the L1 media preparation was from the Gulf of Maine, 0.22 μM filtered, and autoclaved prior to nutrient amendment. Cultures were not axenic, but aseptic technique was used to limit the addition of microorganisms from any source other than bloom filtrate.

The experiment lasted 15 days, each day *K. brevis* incubations were monitored for changes in growth using an 10AU Field Fluorometer ﻿(Turner Designs, San Jose, California). On day 15, the bacterial and viral population abundance was monitored using a BioRad ZE5 cell analyzer equipped with a full violet light 405-nm and blue light 488-nm laser capable of measuring green fluorescence as previously described [18, 19]. Giant virus-like populations were present in the VF lysed cultures. At day 15, the fresh VF lysates were used to reinoculated triplicate *K. brevis* cultures CCMP2820 and CCMP2281, following the same method described above. In this third repropagation round, both *K. brevis* strains began declining at day 8 with no fluorescence signal observed by day 11. On day 11, 3 mL from each biological replicate was flash frozen in liquid nitrogen and stored at -80°C until DNA could be extracted following the same protocol described above. Extracted DNA was used for subsequent GVMAG vOTU1-6 PCR analyses.

Primer design for giant virus vOTU1-6

End point polymerase chain reaction (PCR) primers were designed for the major capsid protein gene of each giant virus vOTU using Primer3 [20], in silico tested using PCR Primer Stat [9], and specificity was tested by searching each primer against the NCBI nt database using NCBI-Primer blast (https://www.ncbi.nlm.nih.gov/tools/primer-blast/). PCR analyses were done using either a Bio-Rad T100 Thermal Cycler or a Bio-Rad C1000 Touch Thermal Cycler with initial denaturing at 95°C for 5 minutes, each cycle included denaturing at 95°C for 30 seconds, annealing for 45 seconds at the temperature listed for each primer set in Supplemental Table 10, and extension at 72°C for 60 seconds. After 40 rounds of amplification the final extension was done at 72°C for an additional 5 minutes before the PCR products were held at 15°C and frozen. Each PCR reaction was done in 24µL and contained 0.25µM each of Forward and Reverse primer, 12.5µL of 2x GoTaq Green Master Mix, 4µL PCR-grade water, and 8µL of extracted sample. PCR products were loaded onto a 1% agarose gel made with 1X sodium borate buffer and gel electrophoresis was run at 90V for 105 minutes. Gels were stained with GelRed for 1 hour followed by a 15-minute destain in DI water. Stained gels were imaged under UV light with an ethidium bromide emission filter using a UVP Gel Doc-It^2^ Imager transilluminator. Of the five primer pairs, primers 1-2 and 13-14 had observable amplified MCP product bands.

PCR products amplified from the positive control and the viral propagation experiments was cleaned following the standard directions of an Omega Bio-Tex E.Z.N.A Cycle Pure Kit. Samples were submitted to the Bioanalytical Services Laboratory at the Institute of Marine and Environmental Technology for GridION, Oxford Nanopore sequencing. Resulting fastq files were aligned to vOTU1-6 genomes using the EPI2ME wf-alignment (https://github.com/epi2me-labs/wf-alignment).

Identification of *Aquintoviricetes* (polinton-like viruses)

All assembled contigs from the five enrichment experiment metagenomes were searched using ICTV_VirophageSG (<https://github.com/simroux/ICTV_VirophageSG>), all returned hits matched to PC_054, a pATPase known to be carried conserved in polinton-like virus (PLV) [21, 22]. Open reading frames (ORF) were predicted for all assembled contigs from the five enrichment metagenomes using pyrodigal-gv v.3.5.1 [23]. All predicted open reading frames were interrogated using previously built major capsid protein (MCP) Hidden Markov Models (HMM) using hmmer v.3.3.2 (hmmsearch --noali -E 0.001) [10]. Contigs that contained a PLV MCP with a bit score of 50 or higher were extracted, aligned with previously reported PLV MCP using mafft v7.487 (--auto), trimmed using trimal v1.4.22 (-automated1), to build a PLV MCP phylogenetic tree using iq-tree v2.2.2.6 (-m MFP -bb 1000 -nt AUTO -wbt) [13, 14, 22, 24]. All ORFs on contigs that contained both PLV MCP and pATPase were annotated using HHpred [25]. Assembled contigs from EH25 metagenomes were searched for evidence of PLV using the methods described above. Only a single contig from the September 2021 metagenome contained both the PC_054 ATPase and MCP.

The average number of aligned reads overlapping each position on the PLV contigs from the five viral enrichment experiment metagenomes were recruited using coverm v0.7.0 using options --min-read-percent-identity 90 --min-read-aligned-percent 75 --min-covered-fraction 0 --minimap2-parameters "-I 500M" (<https://github.com/wwood/CoverM?tab=readme-ov-file#coverm>). Coverm average PLV recruited reads were graphed in R using ggplot2.

Statistical Analyses

All *p*-values were calculated in R using the R Stats package [26]. The pairwise.t.test function with p.adjust.method = “none” was used to calculate F*_v_*/F*_m_*, VLP, and PLV1 significant differences. The output table was generated using the R library rempsyc [27].Correlation analyses between *K. brevis* concentration and vOTU relative abundance at EH25 were calculated and plotted in R using ggplot2 and ggpmisc using stat_poly_line() and stat_poly_eq() [28, 29].

**References**

1. Guillard RRL, Hargraves PE. *Stichochrysis immobilis* is a diatom, not a chrysophyte. *Phycol*. 1993;

2. Bushnell B. BBMap: A Fast, Accurate, Splice-Aware Aligner. 2014.

3. Li H. BFC: correcting Illumina sequencing errors. *Bioinformatics*. 2015; https://doi.org/10.1093/bioinformatics/btv290.

4. Luo E, Eppley JM, Romano AE, Mende DR, DeLong EF. Double-stranded DNA virioplankton dynamics and reproductive strategies in the oligotrophic open ocean water column. *ISME J*. 2020; https://doi.org/10.1038/s41396-020-0604-8.

5. Nurk S, Meleshko D, Korobeynikov A, Pevzner PA. metaSPAdes: a new versatile metagenomic assembler. *Genome Res*. 2017; https://doi.org/10.1101/gr.213959.116.

6. Danecek P, Bonfield JK, Liddle J, Marshall J, Ohan V, Pollard MO, et al. Twelve years of SAMtools and BCFtools. *Gigascience*. 2021; https://doi.org/10.1093/gigascience/giab008.

7. Kang DD, Froula J, Egan R, Wang Z. MetaBAT, an efficient tool for accurately reconstructing single genomes from complex microbial communities. *PeerJ*. 2015; https://doi.org/10.7717/peerj.1165.

8. Pitot TM, Brůna T, Schulz F. Conservative taxonomy and quality assessment of giant virus genomes with GVClass. *npj Viruses*. 2024; https://doi.org/10.1038/s44298-024-00069-7.

9. Nayfach S, Camargo AP, Schulz F, Eloe-Fadrosh E, Roux S, Kyrpides NC. CheckV assesses the quality and completeness of metagenome-assembled viral genomes. *Nat Biotechnol*. 2021; https://doi.org/10.1038/s41587-020-00774-7.

10. Camargo AP, Roux S, Schulz F, Babinski M, Xu Y, Hu B, et al. Identification of mobile genetic elements with geNomad. *Nat Biotechnol*. 2023; https://doi.org/10.1038/s41587-023-01953-y.

11. Moniruzzaman M, Martinez-Gutierrez CA, Weinheimer AR, Aylward FO. Dynamic genome evolution and complex virocell metabolism of globally-distributed giant viruses. *Nat Commun*. 2020; https://doi.org/10.1038/s41467-020-15507-2.

12. Aylward FO, Moniruzzaman M, Ha AD, Koonin E V. A phylogenomic framework for charting the diversity and evolution of giant viruses. *PLOS Biol*. 2021; https://doi.org/10.1371/journal.pbio.3001430.

13. Capella-Gutiérrez S, Silla-Martínez JM, Gabaldón T. trimAl: a tool for automated alignment trimming in large-scale phylogenetic analyses. *Bioinformatics*. 2009; https://doi.org/10.1093/bioinformatics/btp348.

14. Minh BQ, Schmidt HA, Chernomor O, Schrempf D, Woodhams MD, von Haeseler A, et al. IQ-TREE 2: New Models and Efficient Methods for Phylogenetic Inference in the Genomic Era. *Mol Biol Evol*. 2020; https://doi.org/10.1093/molbev/msaa015.

15. Stamatakis A. RAxML version 8: a tool for phylogenetic analysis and post-analysis of large phylogenies. *Bioinformatics*. 2014; https://doi.org/10.1093/bioinformatics/btu033.

16. Fei C, Booker A, Klass S, Vidyarathna NK, Ahn SH, Mohamed AR, et al. Friends and foes: symbiotic and algicidal bacterial influence on Karenia brevis blooms. *ISME Commun*. 2025; https://doi.org/10.1093/ismeco/ycae164.

17. Cui X, Lu Z, Wang S, Jing-Yan Wang J, Gao X. CMsearch: simultaneous exploration of protein sequence space and structure space improves not only protein homology detection but also protein structure prediction. *Bioinformatics*. 2016; https://doi.org/10.1093/bioinformatics/btw271.

18. Brussaard CPD. Optimization of Procedures for Counting Viruses by Flow Cytometry. *Appl Environ Microbiol*. 2004; https://doi.org/10.1128/AEM.70.3.1506-1513.2004.

19. Marie D, Partensky F, Vaulot D, Brussaard C. Enumeration of Phytoplankton, Bacteria, and Viruses in Marine Samples. *Curr Protoc Cytom*. 1999; https://doi.org/https://doi.org/10.1002/0471142956.cy1111s10.

20. Untergasser A, Cutcutache I, Koressaar T, Ye J, Faircloth BC, Remm M, et al. Primer3—new capabilities and interfaces. *Nucleic Acids Res*. 2012; https://doi.org/10.1093/nar/gks596.

21. Bellas CM, Sommaruga R. Polinton-like viruses are abundant in aquatic ecosystems. *Microbiome*. 2021; https://doi.org/10.1186/s40168-020-00956-0.

22. Bellas C, Hackl T, Plakolb M-S, Koslová A, Fischer MG, Sommaruga R. Large-scale invasion of unicellular eukaryotic genomes by integrating DNA viruses. *Proc Natl Acad Sci*. 2023; https://doi.org/10.1073/pnas.2300465120.

23. Clément C, Pedro CA, Simon R. MVP: a modular viromics pipeline to identify, filter, cluster, annotate, and bin viruses from metagenomes. *mSystems*. 2024; https://doi.org/10.1128/msystems.00888-24.

24. Nakamura T, Yamada KD, Tomii K, Katoh K. Parallelization of MAFFT for large-scale multiple sequence alignments. *Bioinformatics*. 2018; https://doi.org/10.1093/bioinformatics/bty121.

25. Zimmermann L, Stephens A, Nam SZ, Rau D, Kübler J, Lozajic M, et al. A Completely Reimplemented MPI Bioinformatics Toolkit with a New HHpred Server at its Core. *J Mol Biol*. 2018; https://doi.org/10.1016/j.jmb.2017.12.007.

26. R Core Team. R: A Language and Environment for Statistical Computing. 2025. R Foundation for Statistical Computing, Vienna, Austria.

27. Thériault R. rempsyc : Convenience functions for psychology. *J Open Source Softw*. 2023; https://doi.org/10.21105/joss.05466.

28. Wickham H. ggplot2: Elegant Graphics for Data Analysis. 2016. Springer-Verlag New York.

29. Aphalo PJ. ggpmisc: Miscellaneous Extensions to “ggplot2.” 2025. https://doi.org/10.32614/CRAN.package.ggpmisc.

**Supplemental Figures**

**
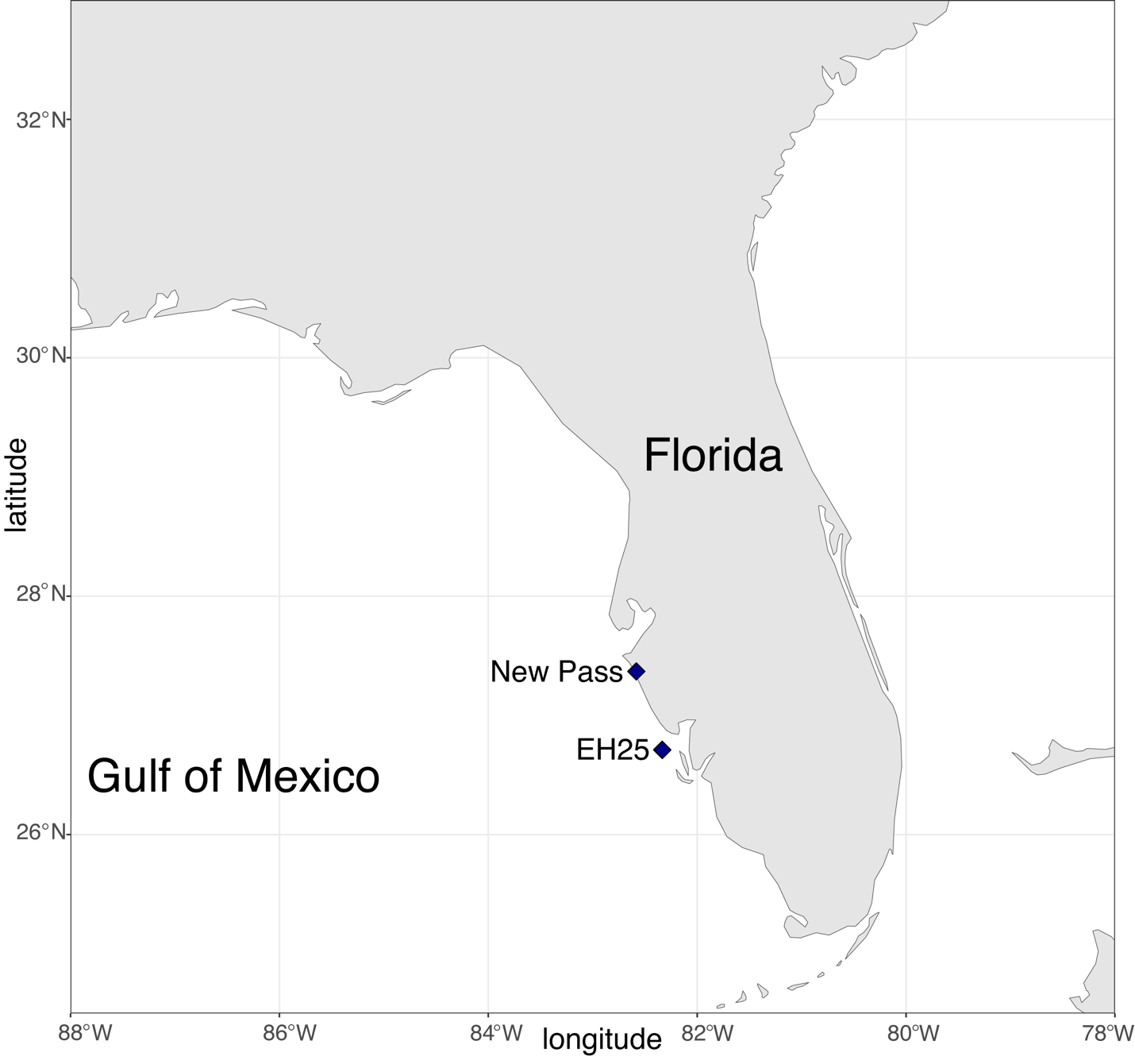
**

**Supplemental Figure 1. Sampling Sites.** Geographical location of the monthly sampling station EH25 relative to the New Pass sampling site.

**
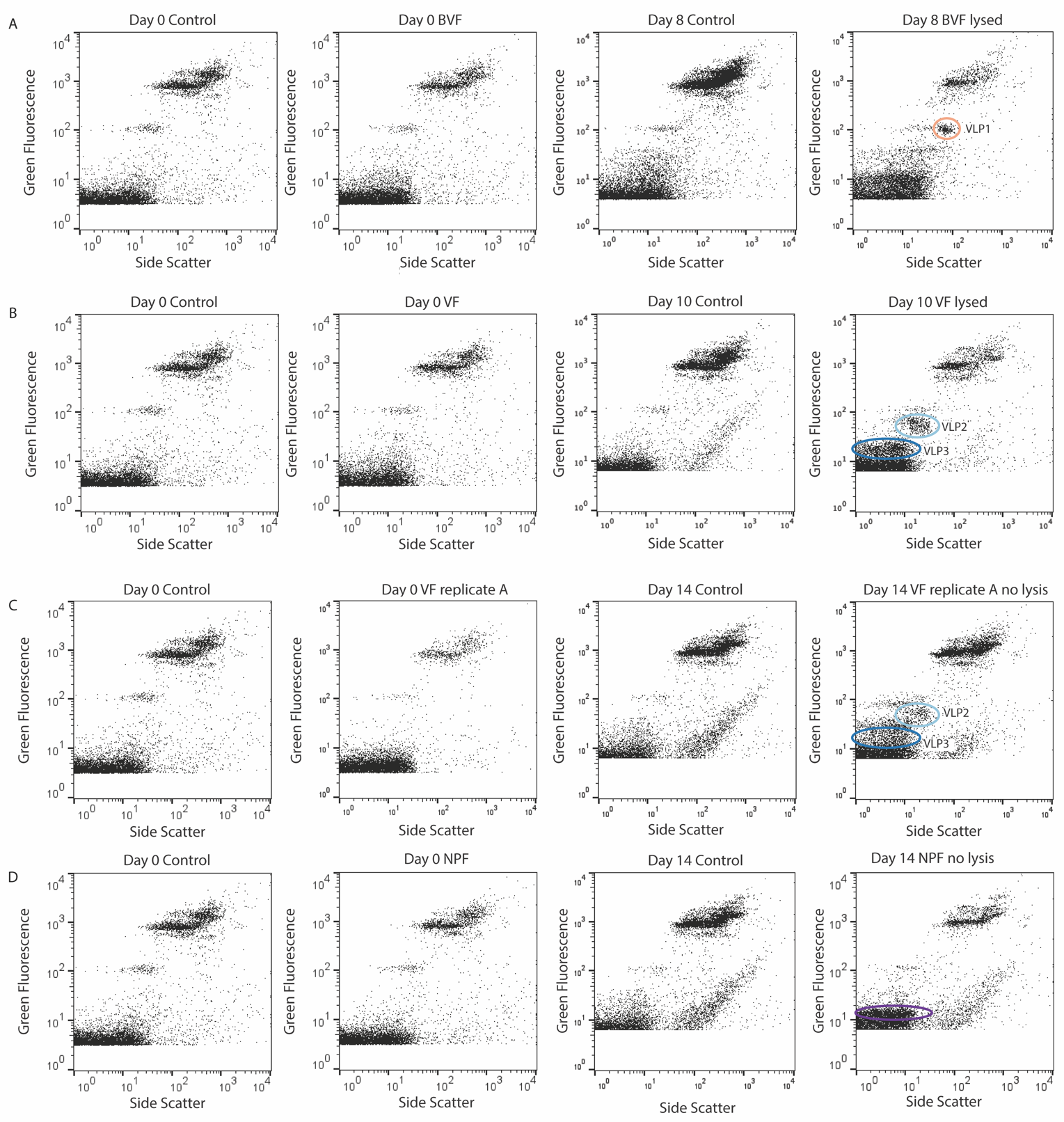
**

**Supplemental Figure 2. Representative flow cytometry plots used to visualize viral-like populations (VLP) in the incubation flasks.** Representative plots were chosen from the beginning of the experiment (day 0) and days when VLPs, absent in the control cultures, were detected in the BVF (**A**), VF (**B**, **C**), or NPF (**D**) treatment cultures, irrespective of the *F_v_/F_m_* values measured. Presumed giant VLPs (based on their relatively high green fluorescence and side scatter signals) were identified in the BVF cultures (VLP1, peach ellipse) and the VF cultures (VLP2, light blue ellipse; VLP3 dark blue ellipse). A VLP with relatively lower green fluorescence and side scatter signals (purple ellipse) was detected in the NPF cultures by day 14 of the incubation.


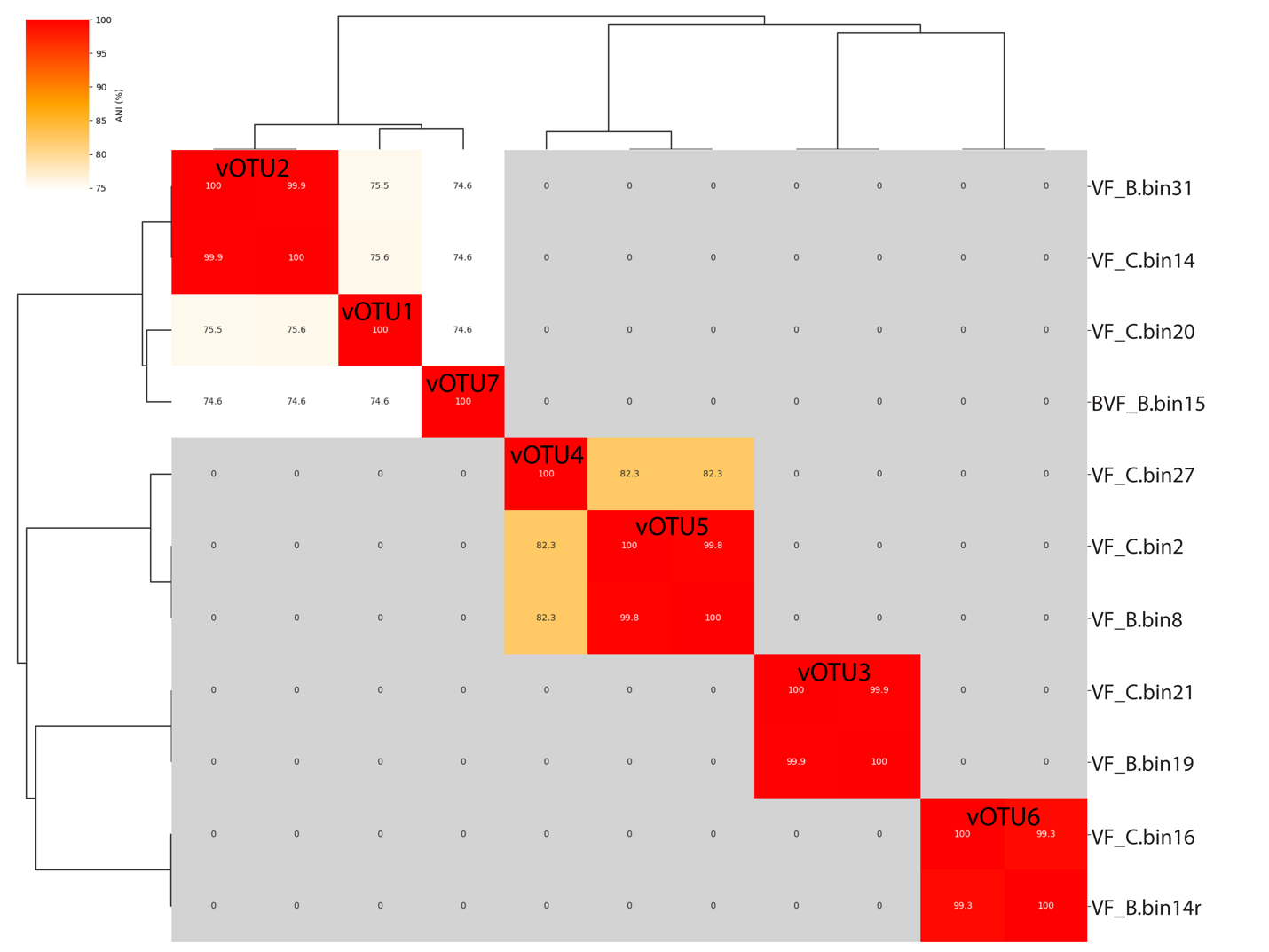


**Supplemental Figure 3. Heatmap of average nucleotide identity (ANI) for the eleven GVMAGs assembled from the incubation experiment metagenomes.** GVMAG identifiers are listed along the right Y-axis. Values range from 0 (0%, grey) ANI to 100 (100%, red) ANI. GVMAGs with over 95% ANI (and 85% minimum alignment fraction) represent viral operational taxonomic units (vOTUs).

**
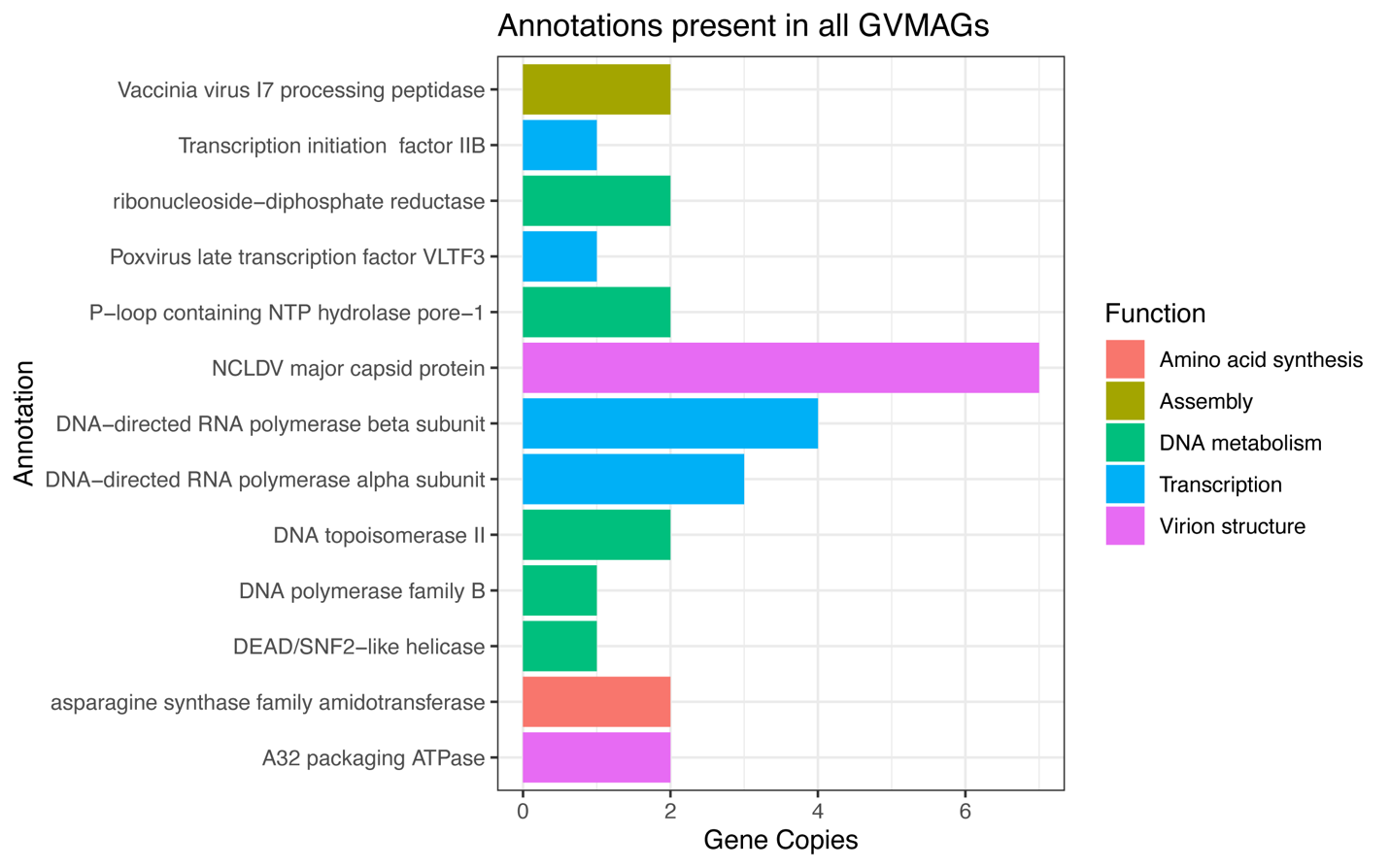
**

**Supplemental Figure 4. List of conserved genes across all the VF GVMAGs.** The maximum number of gene copies found in an individual GVMAG is represented on the x-axis. Bars are colored based on their conserved function.


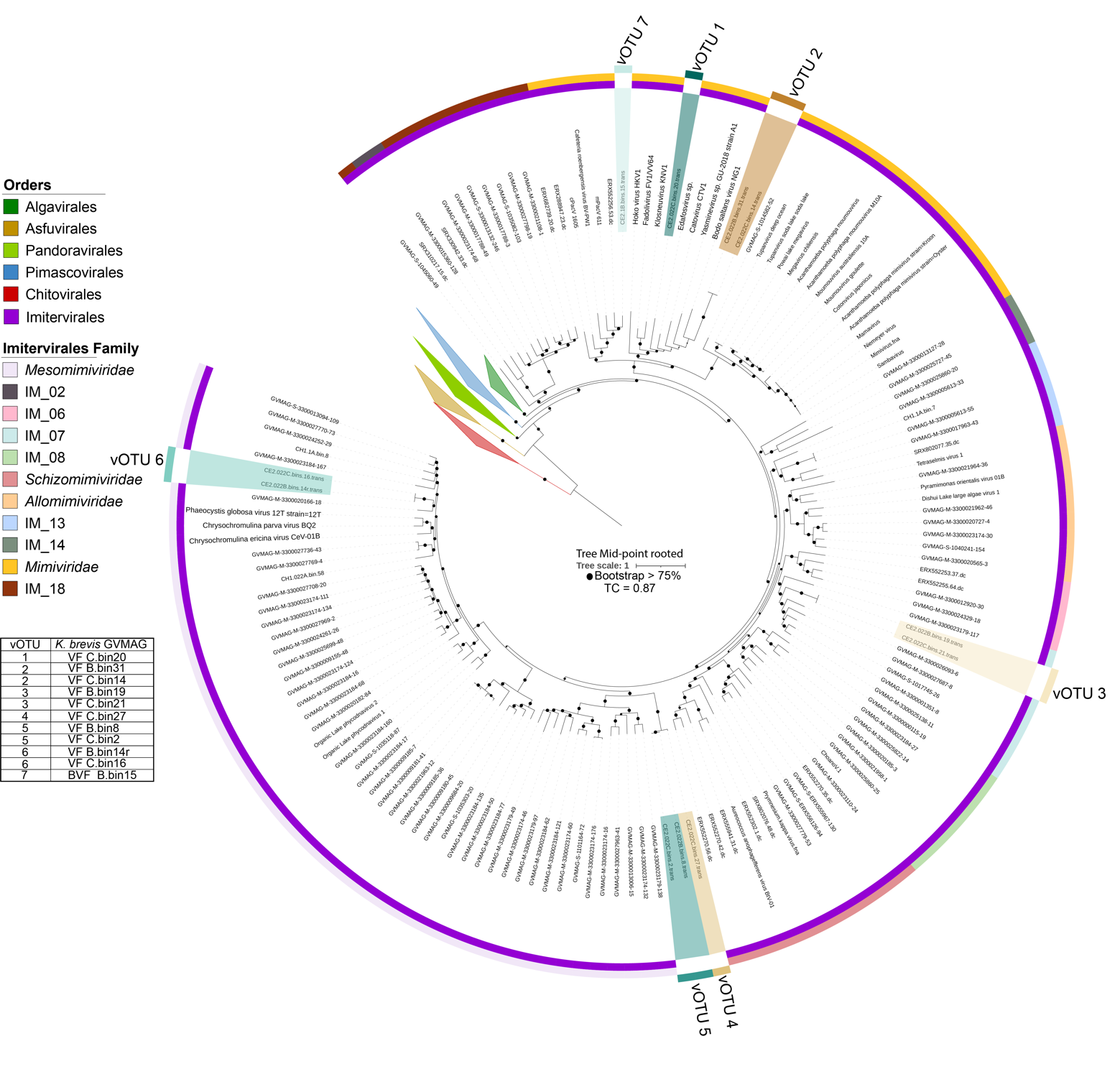


**Supplemental Figure 5.** **Mid-point rooted *Nucleocytoviricota* conserved marker gene phylogenomic tree showing vOTU1-7 taxonomic affilitations.** Bootstrap values were calculated based on 1000 trees and branch support above 75% is represented by black circles. The tree certainty (TC) value is 87%. All GVMAGs identified in this study fall within the order *Imitervirales* (inner dark purple ring). Collapsed clades represent other *Nucleocytoviricota* orders. Outer ring colors denote families within the order *Imitervirales*. Branches with GVMAGs representing each vOTU (listed in bottom left table) are shaded with different colors. Individual genome identifiers are given for each tree branch and listed in Supplemental Data 8.


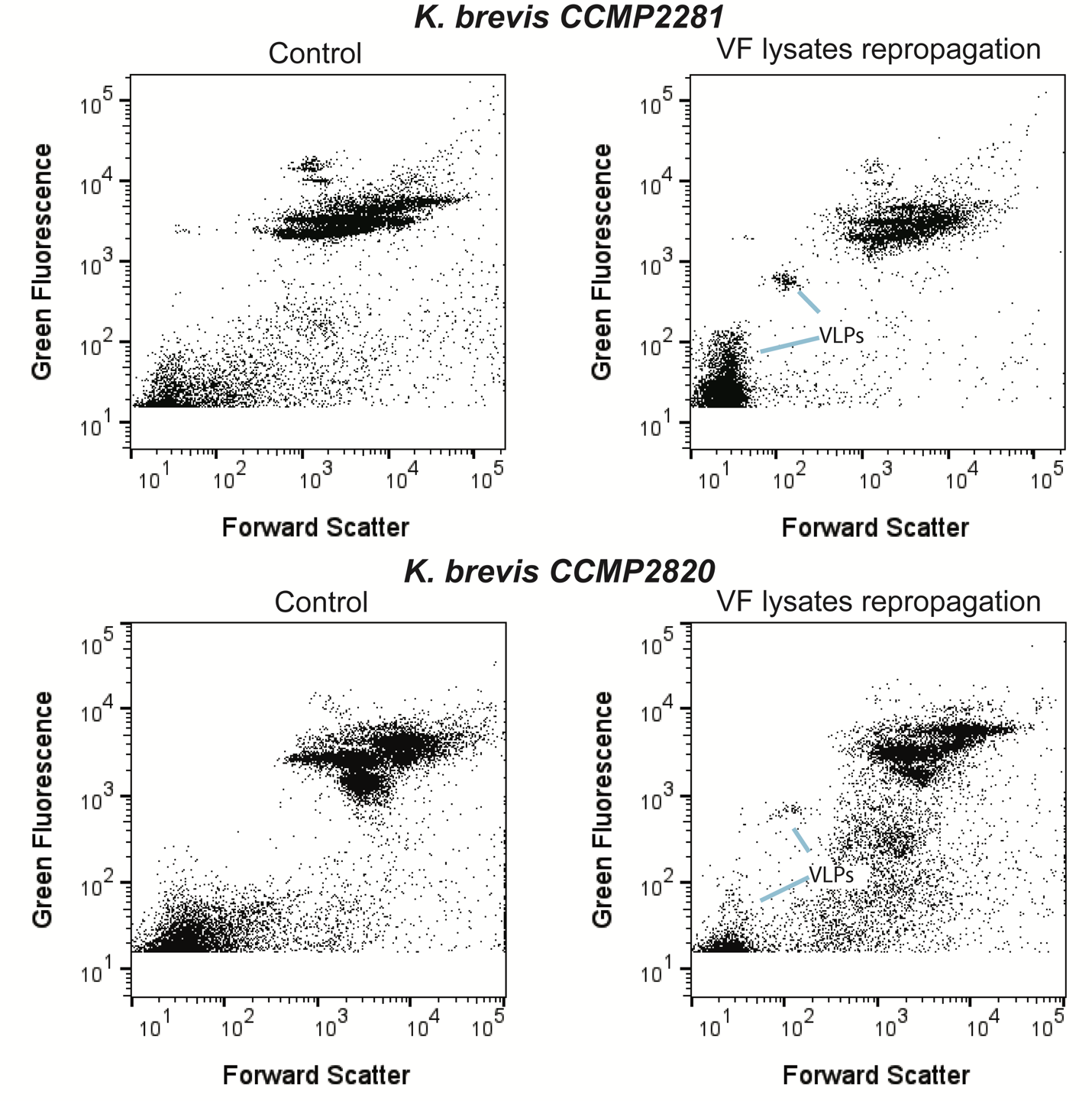


**Supplemental Figure 6. Representative flow cytometry plots from the VF lysates repropagation experiment using *K. brevis* strains CCMP2281 and CCMP2820.** Selected plots show distinct virus-like populations (VLPs) present in the lysed cultures (right panels) but not in the respective non-inoculated control cultures (left panels).


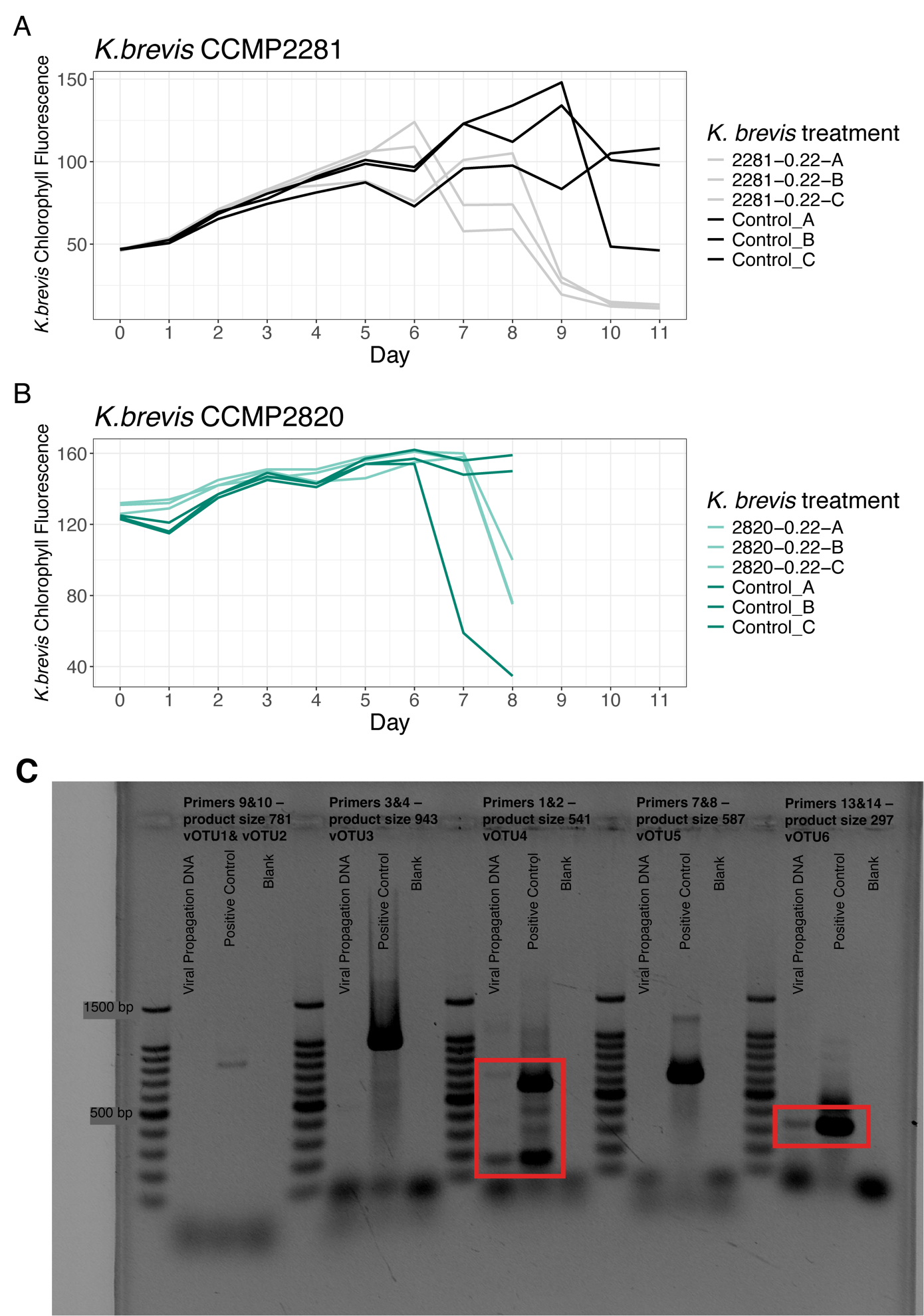


**Supplemental Figure 7. Repropagation of *K. brevis* associated giant viruses using *K. brevis* strains CCMP2281 and CCMP2820.** **A)** Fluorescence levels in replicate cultures of *K. brevis* CCMP2281: non-inoculated controls (black lines) and inoculated with lysates originated from the bloom water incubation experiment (grey lines); **B)** Fluorescence levels in replicate cultures of *K. brevis* CCMP2820: non-inoculated controls (dark green lines) and inoculated with lysates originated from the bloom water incubation experiment (light green lines); **C)** Gel image of end-point PCRs products using vOTU-specific primer sets on DNA extracted from the combined resulting lysates from CCMP2281 and CCMP2820. Red rectangles highlight positive amplification, based on the presence of bands with the expected amplicon size compared to positive PCR controls. Columns 1, 5, 9, 13, and 17 contain DM2100 ExcelBand 100 bp DNA ladder (Stellar Scientific, MD, USA). Columns marked as “Blank” were loaded with no-amplification PCR controls that contained water instead of DNA template.

**
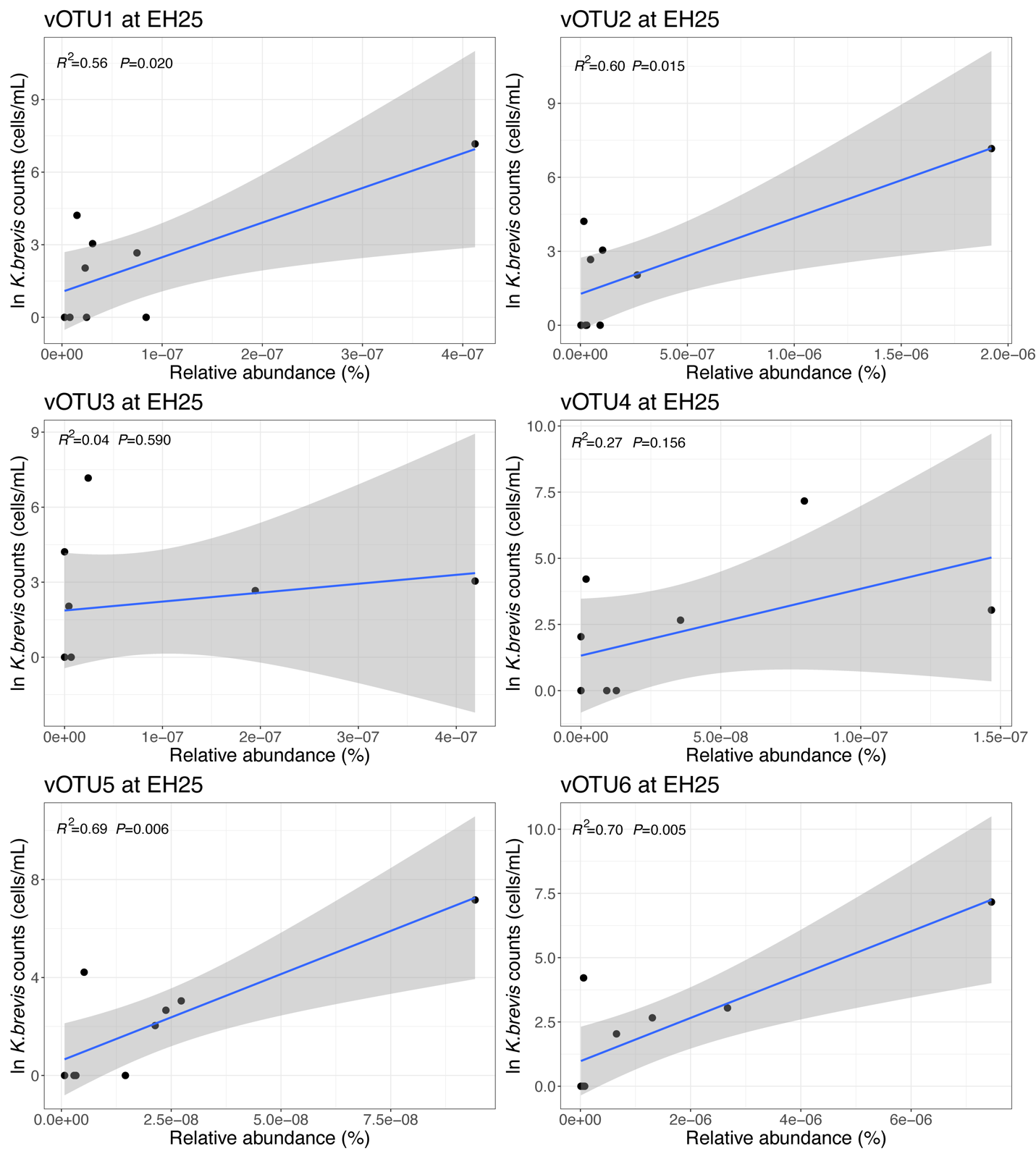
**

**Supplemental Figure 8. Correlation analysis between *K. brevis* concentration and giant virus (vOTU) relative abundance at sampling location EH25.** Blue line represents the linear regression fit to the data. Gray area represents the 95% confidence interval.

**
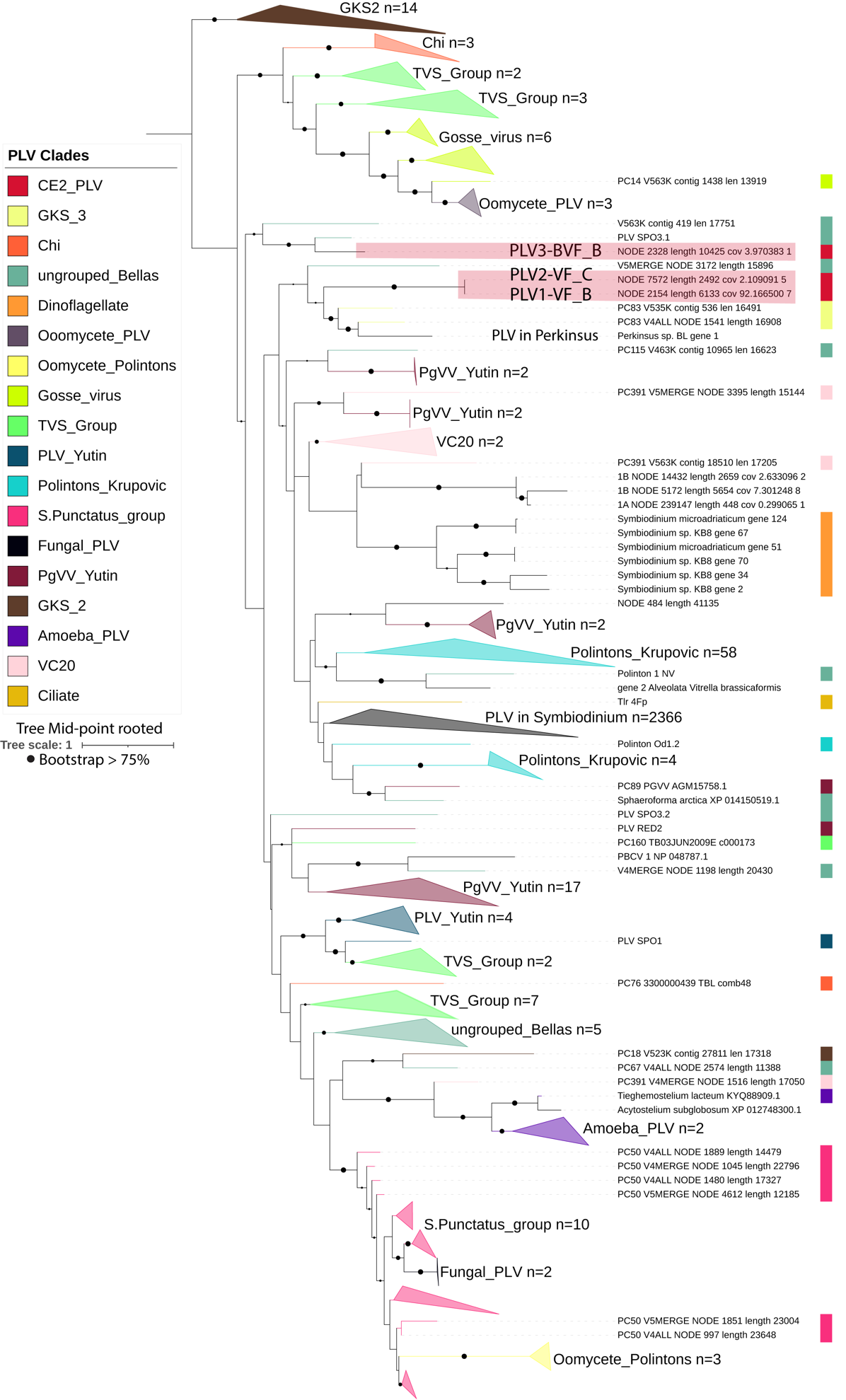
**

**Supplemental Figure 9. Polinton-like virus major capsid protein (MCP) gene phylogenetic tree.** The colors of the collapsed clades and on the right hand side of the tree correspond to PLV clades based on MCP gene sequences from previous network analyses (Bellas et al., 2021, 2023). The tree is mid-point rooted in iTOL. Bootstrap values are calculated based on 1000 trees and branch support above 75% is represented by black circles. PLV sequences included in this tree are listed in Supplemental Data File 17.


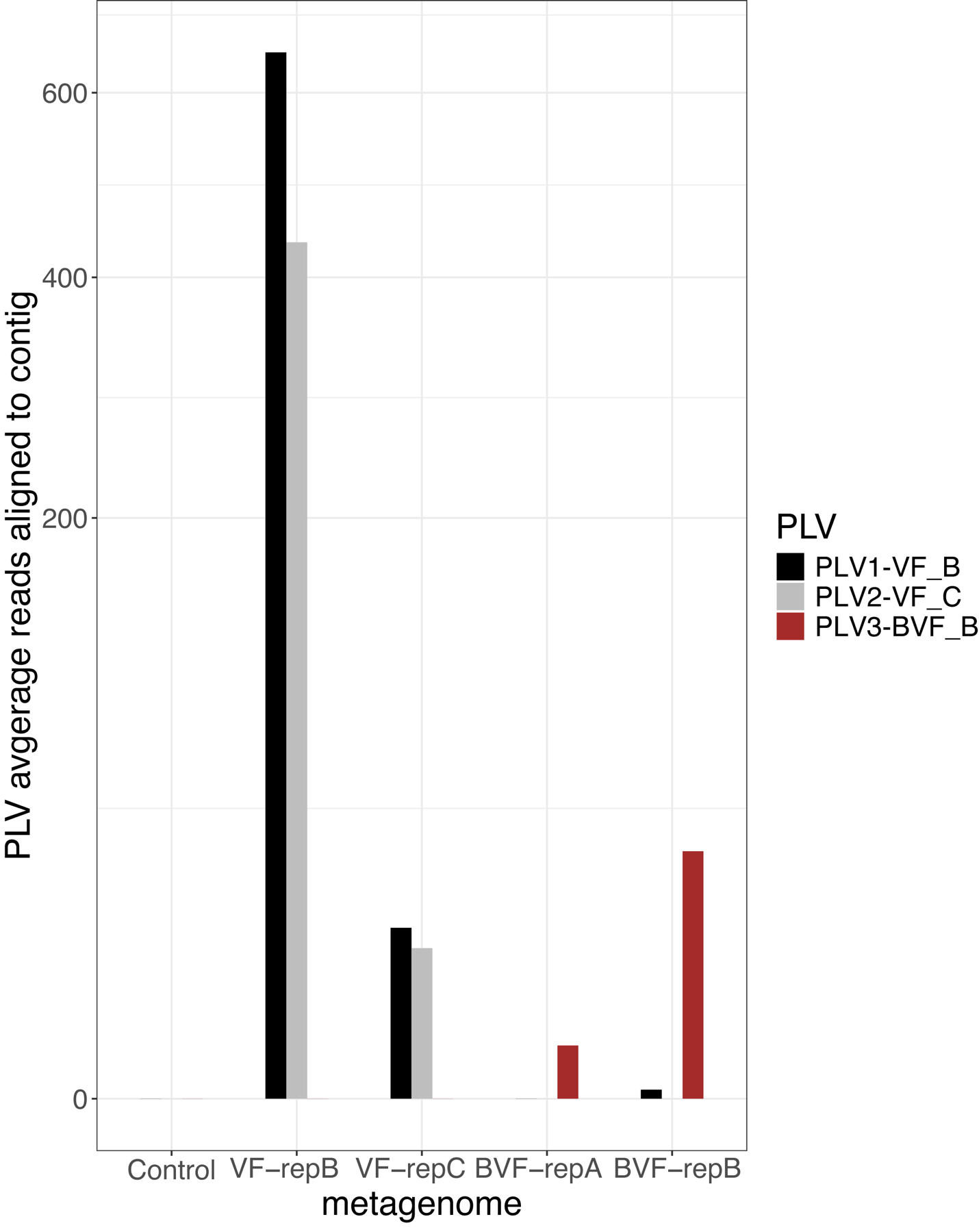


**Supplemental Figure 10.** The average number of reads from the incubation experiment metagenomes that mapped to each PLV contig. PLV contigs must contain the two maker genes, major capsid protein (MCP) and FtsK-HerA ATPase.

**Supplemental Tables**

Supplemental Table 1. Pairwise T Test comparing the F*_v_*/F*_m_* values of *K. brevis* biological triplicates treated with 1uM filtered seawater, 0.2uM filtered seawater, or no seawater control.

| Treatment 1 | Treatment 2 | *p* |
| --- | --- | --- |
| 0.2uM | 0.02uM | .006** |
| 1uM | 0.02uM | < .001*** |
| 1uM | 0.2uM | .426 |
| control | 0.02uM | .734 |
| control | 0.2uM | .013* |
| control | 1uM | .001** |

Supplemental Table 2. Pairwise T Test comparing Viral-like population (1,2,3) concentrations across nine *K. brevis* incubations.

vs.

| incubation | incubation | *p* |
| --- | --- | --- |
| VLP1_1uM_5 | VLP1_1uM_4 | .760 |
| VLP1_1uM_6 | VLP1_1uM_4 | .877 |
| VLP1_1uM_6 | VLP1_1uM_5 | .645 |
| VLP1_control_1 | VLP1_1uM_4 | .768 |
| VLP1_control_1 | VLP1_1uM_5 | .995 |
| VLP1_control_1 | VLP1_1uM_6 | .659 |
| VLP1_control_2 | VLP1_1uM_4 | .768 |
| VLP1_control_2 | VLP1_1uM_5 | .995 |
| VLP1_control_2 | VLP1_1uM_6 | .659 |
| VLP1_control_2 | VLP1_control_1 | 1.00 |
| VLP1_control_3 | VLP1_1uM_4 | .768 |
| VLP1_control_3 | VLP1_1uM_5 | .995 |
| VLP1_control_3 | VLP1_1uM_6 | .659 |
| VLP1_control_3 | VLP1_control_1 | 1.00 |
| VLP1_control_3 | VLP1_control_2 | 1.00 |
| VLP2_0.2uM_8 | VLP2_0.2uM_7 | .346 |
| VLP2_0.2uM_9 | VLP2_0.2uM_7 | .529 |
| VLP2_0.2uM_9 | VLP2_0.2uM_8 | .753 |
| VLP2_control_1 | VLP2_0.2uM_7 | .613 |
| VLP2_control_1 | VLP2_0.2uM_8 | .149 |
| VLP2_control_1 | VLP2_0.2uM_9 | .257 |
| VLP2_control_2 | VLP2_0.2uM_7 | .613 |
| VLP2_control_2 | VLP2_0.2uM_8 | .149 |
| VLP2_control_2 | VLP2_0.2uM_9 | .257 |
| VLP2_control_2 | VLP2_control_1 | 1.00 |
| VLP2_control_3 | VLP2_0.2uM_7 | .613 |
| VLP2_control_3 | VLP2_0.2uM_8 | .149 |
| VLP2_control_3 | VLP2_0.2uM_9 | .257 |
| VLP2_control_3 | VLP2_control_1 | 1.00 |
| VLP2_control_3 | VLP2_control_2 | 1.00 |
| VLP3_0.2uM_8 | VLP3_0.2uM_7 | .003** |
| VLP3_0.2uM_9 | VLP3_0.2uM_7 | .244 |
| VLP3_0.2uM_9 | VLP3_0.2uM_8 | .070 |
| VLP3_control_1 | VLP3_0.2uM_7 | .152 |
| VLP3_control_1 | VLP3_0.2uM_8 | < .001*** |
| VLP3_control_1 | VLP3_0.2uM_9 | .011* |
| VLP3_control_2 | VLP3_0.2uM_7 | .152 |
| VLP3_control_2 | VLP3_0.2uM_8 | <.001*** |
| VLP3_control_2 | VLP3_0.2uM_9 | .011* |
| VLP3_control_2 | VLP3_control_1 | 1.00 |
| VLP3_control_3 | VLP3_0.2uM_7 | .152 |
| VLP3_control_3 | VLP3_0.2uM_8 | <.001*** |
| VLP3_control_3 | VLP3_0.2uM_9 | .011* |
| VLP3_control_3 | VLP3_control_1 | 1.00 |
| VLP3_control_3 | VLP3_control_2 | 1.00 |
| VLP3_0.2uM_9 | VLP1_1uM_4 | .033* |
| VLP3_0.2uM_9 | VLP1_1uM_5 | .015* |
| VLP3_0.2uM_9 | VLP1_1uM_6 | .048* |
| VLP3_0.2uM_8 | VLP1_1uM_4 | <.001*** |
| VLP3_0.2uM_8 | VLP1_1uM_5 | <.001*** |
| VLP3_0.2uM_8 | VLP1_1uM_6 | <.001*** |
| VLP3_0.2uM_7 | VLP1_1uM_4 | .297 |
| VLP3_0.2uM_7 | VLP1_1uM_5 | .174 |
| VLP3_0.2uM_7 | VLP1_1uM_6 | .377 |
|  |  |  |
|  |  |  |
|  |  |  |

Supplemental Table 3. Pairwise T-test comparing the average number of PLV1 aligned reads in each incubation experiment metagenome. Control contains no seawater amendment, BVF replicate A and B contained 1µM filtered seawater, and VF replicate B and C contained 0.22µM filtered seawater. Metagenome VF-B contained a statistically significant concentration of PLV1 aligned reads.

| incubation | incubation | *p* |
| --- | --- | --- |
| VF-C | VF-B | < .001*** |
| BVF-A | VF-B | < .001*** |
| BVF-A | VF-C | .727 |
| BVF-B | VF-B | < .001*** |
| BVF-B | VF-C | .947 |
| BVF-B | BVF-A | .678 |
| control | VF-B | < .001*** |
| control | VF-C | .712 |
| control | BVF-A | .984 |
| control | BVF-B | .663 |

**Supplemental Data Files**

**Supplemental Data 1.** Number of sequenced reads, assembled contigs, binned metagenome assembled genomes (MAGs), giant virus MAGs present in the sequenced metagenomes, and metagenome NCBI accession ID.

**Supplemental Data 2.** *Karenia brevis* F*_v_*/F*_m_* values, cell counts, and calculated growth rates observed during the August 2021 incubation experiment.

**Supplemental Data 3.** Concentration of viral-like populations (VLP1-3) throughout the 15-day *K. brevis* viral enrichment experiment.

**Supplemental Data 4.** Giant virus metagenome assembled genomes (GVMAGs) assembly, conserved marker gene, and taxonomic information.

**Supplemental Data 5.** Sequencing statistics and taxonomy of putative beneficial and algicidal bacterial metagenome assembled genomes (MAGs) found in the incubation experiment metagenomes.

**Supplemental Data 6.** GVMAGs blastn alignment comparison.

**Supplemental Data 7.** GVMAG open reading frames annotations using the GeNomad database.

**Supplemental Data 8.** List of *Imitervirales* genome identifiers and family names used in the GVMAG concatenated phylogenetic tree.

**Supplemental Data 9.** CMsearch eukaryotic 18S identification results from the BVF replicate B metagenome. No other 18S hits were present in the control and VF metagenomes.

**Supplemental Data 10.** Average number of reads from the EH25 station monthly metagenomes aligned to contigs containing the Opalozoa 18S rRNA gene.

**Supplemental Data 11.** PacBio eukaryotic 18S rRNA gene sequencing of *K. brevis* culture strains CCMP2228, CCMP2820, and CCMP2281 used in this study.

**Supplemental Data 12.** End-point PCR primer annealing temperature and primer sequences for the six *K. brevis* vOTU identified in this study.

**Supplemental Data 13.** Read mapping results from Nanopore sequenced vOTU MCP PCR products. The MCP from vOTU5 and vOTU6 were present at a coverage range of 90-46000 in the repropagation experiments.

**Supplemental Data 14.** Phytoplankton cell abundance during monthly sampling at station EH25 and in the bloom sample collected at the Mote New Pass dock for the incubation experiment. (C. Heil, PrimoStar Zeiss Microscope).

**Supplemental Data 15.** Top HHPred results for single ORFs in contigs from the VF and BVF incubation experiment metagenomes that contain the Polinton-like virus markers major capsid proteins (MCP) and FtsK-HerA ATPase.

**Supplemental Data 16.** PLV MCP and FtsK-HerA ATPase HMMsearch summary in coastal Florida EH25 metagenomes.

**Supplemental Data 17.** Polinton-like virus markers major capsid protein sequences used to create the PLV phylogenetic tree. Sequences in fasta format.
