# Supplementary figures and images for: Complex viral interactions revealed for the harmful bloom-forming dinoflagellate Karenia brevis
Source: ISME Commun. 2026 Mar 9;6(1):ycag051. doi: 10.1093/ismeco/ycag051 (PMC13037468; doi:10.1093/ismeco/ycag051)

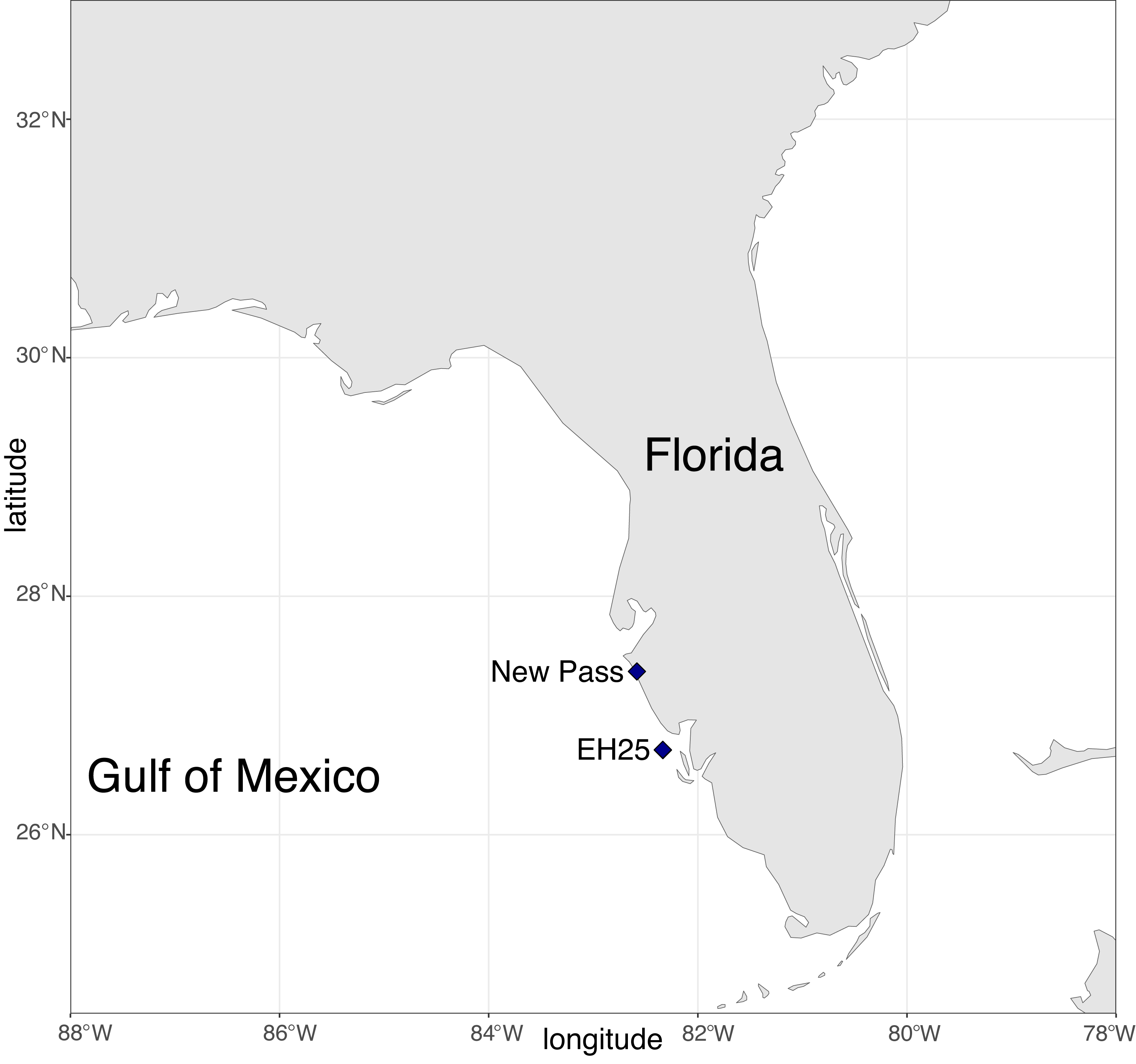

Supplement: ycag051_Supplementary_material [file ycag051_supplementary_material.zip › SupplementalFigure1.tif]

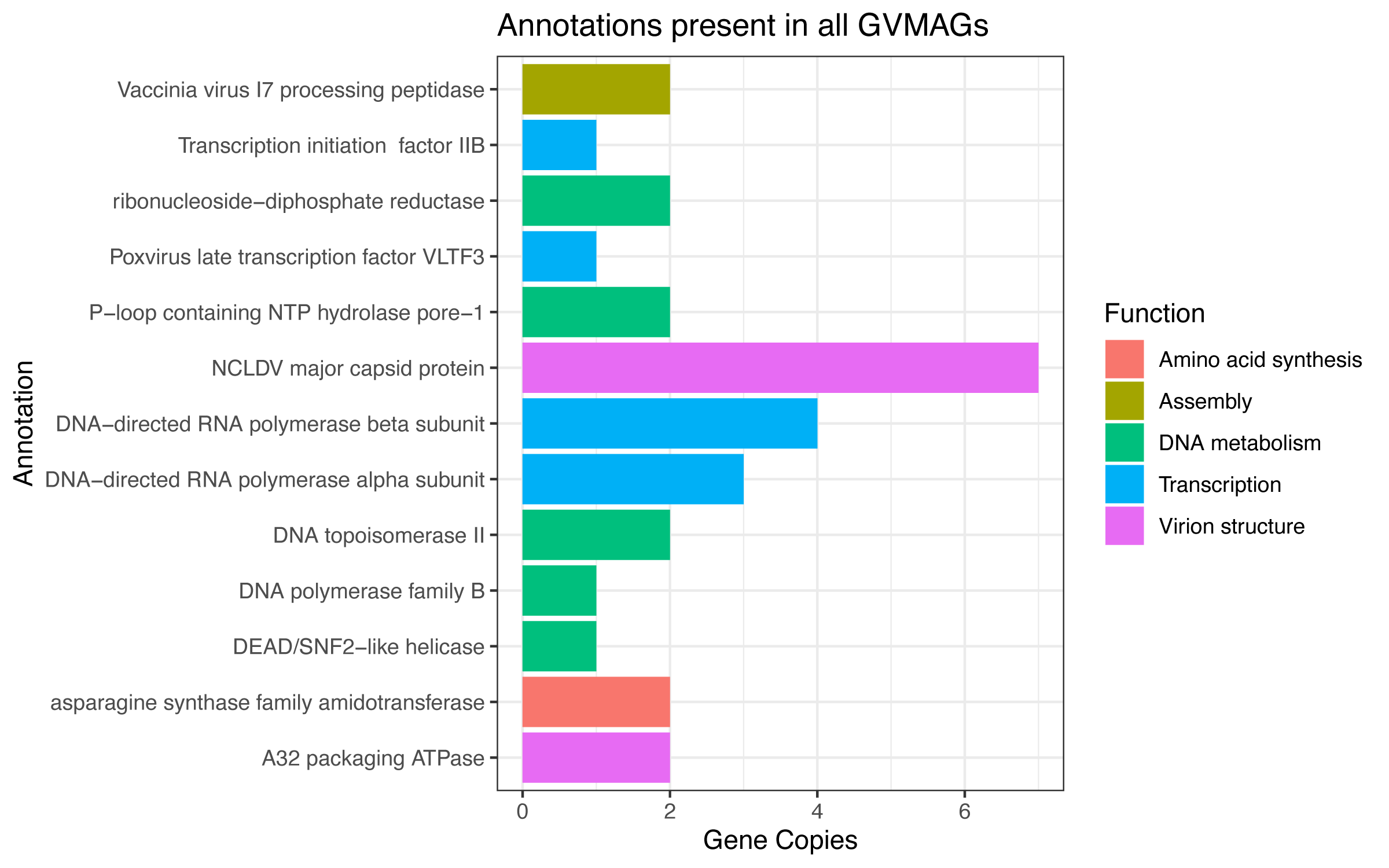

Supplement: ycag051_Supplementary_material [file ycag051_supplementary_material.zip › SupplementalFigure4.tif]

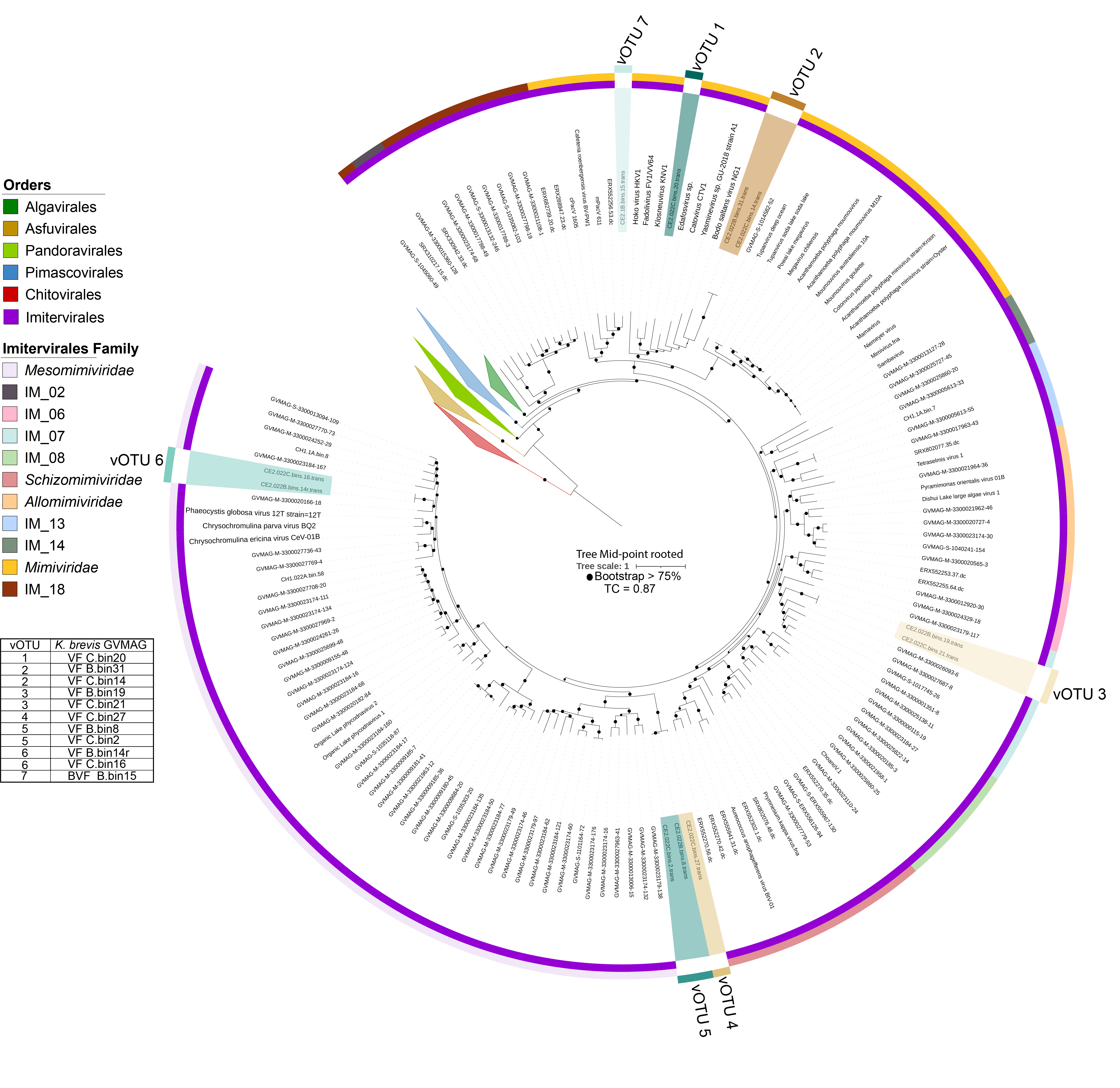

Supplement: ycag051_Supplementary_material [file ycag051_supplementary_material.zip › SupplementalFigure5.png]

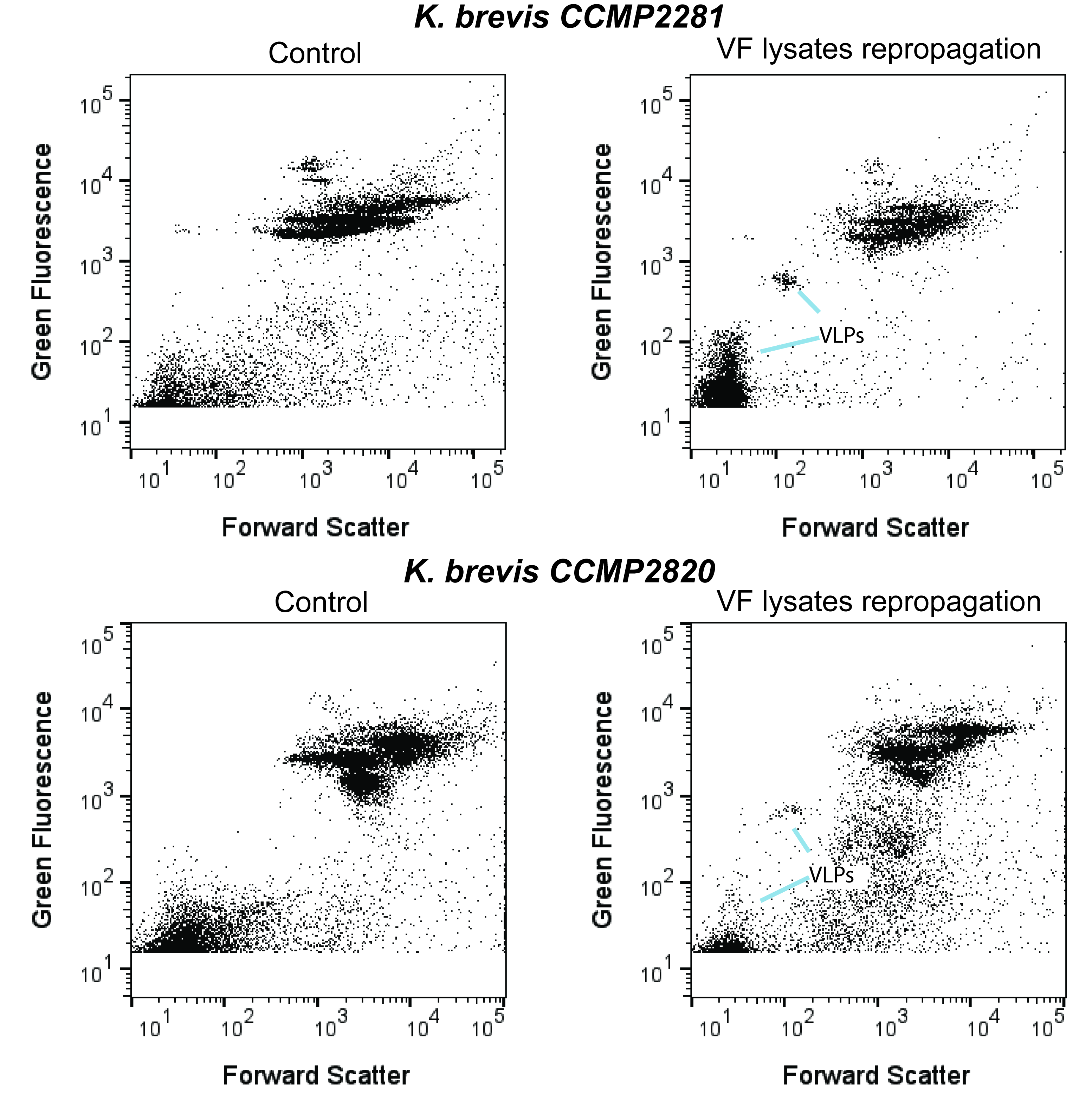

Supplement: ycag051_Supplementary_material [file ycag051_supplementary_material.zip › SupplementalFigure6.tif]

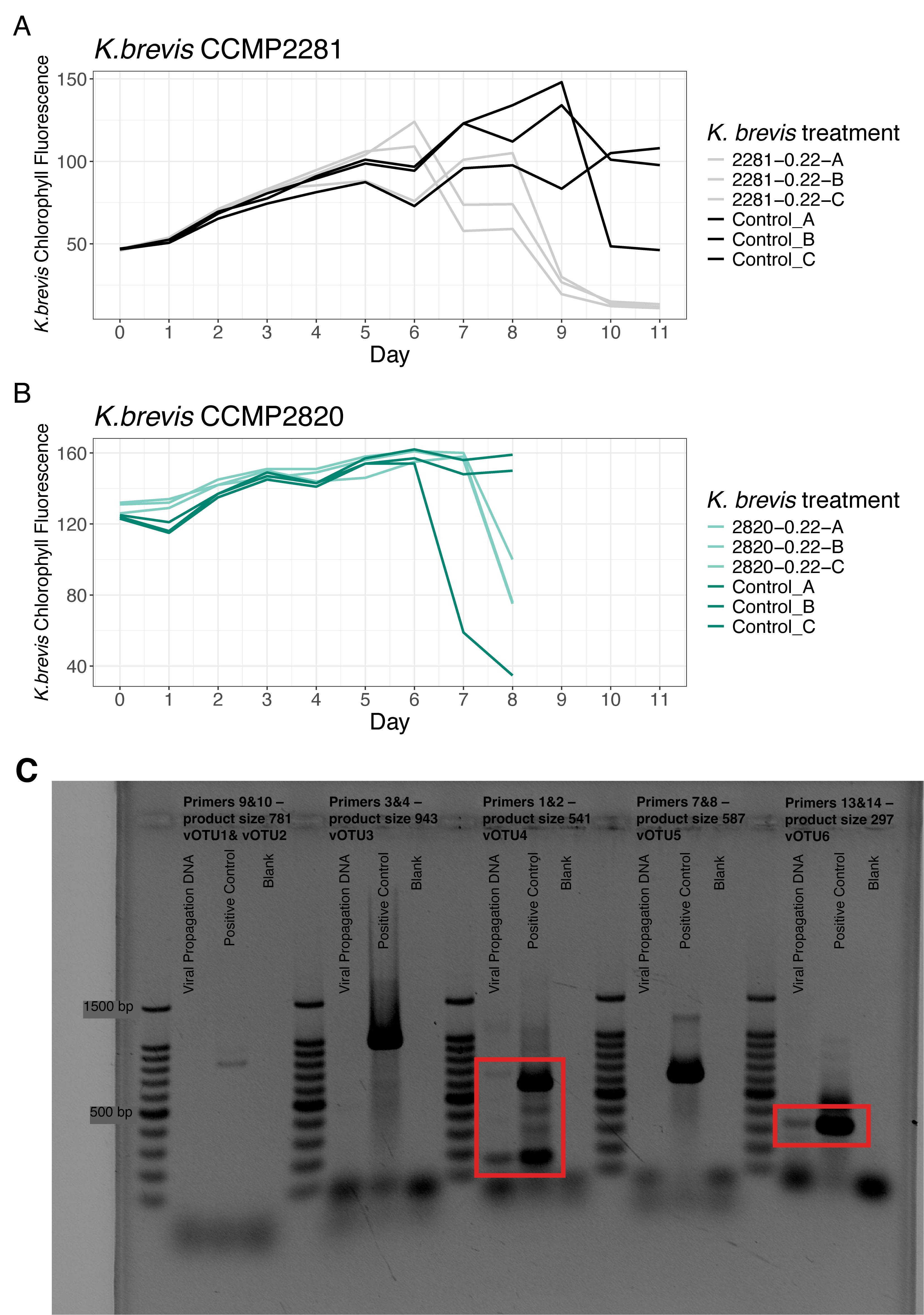

Supplement: ycag051_Supplementary_material [file ycag051_supplementary_material.zip › SupplementalFigure7.tif]

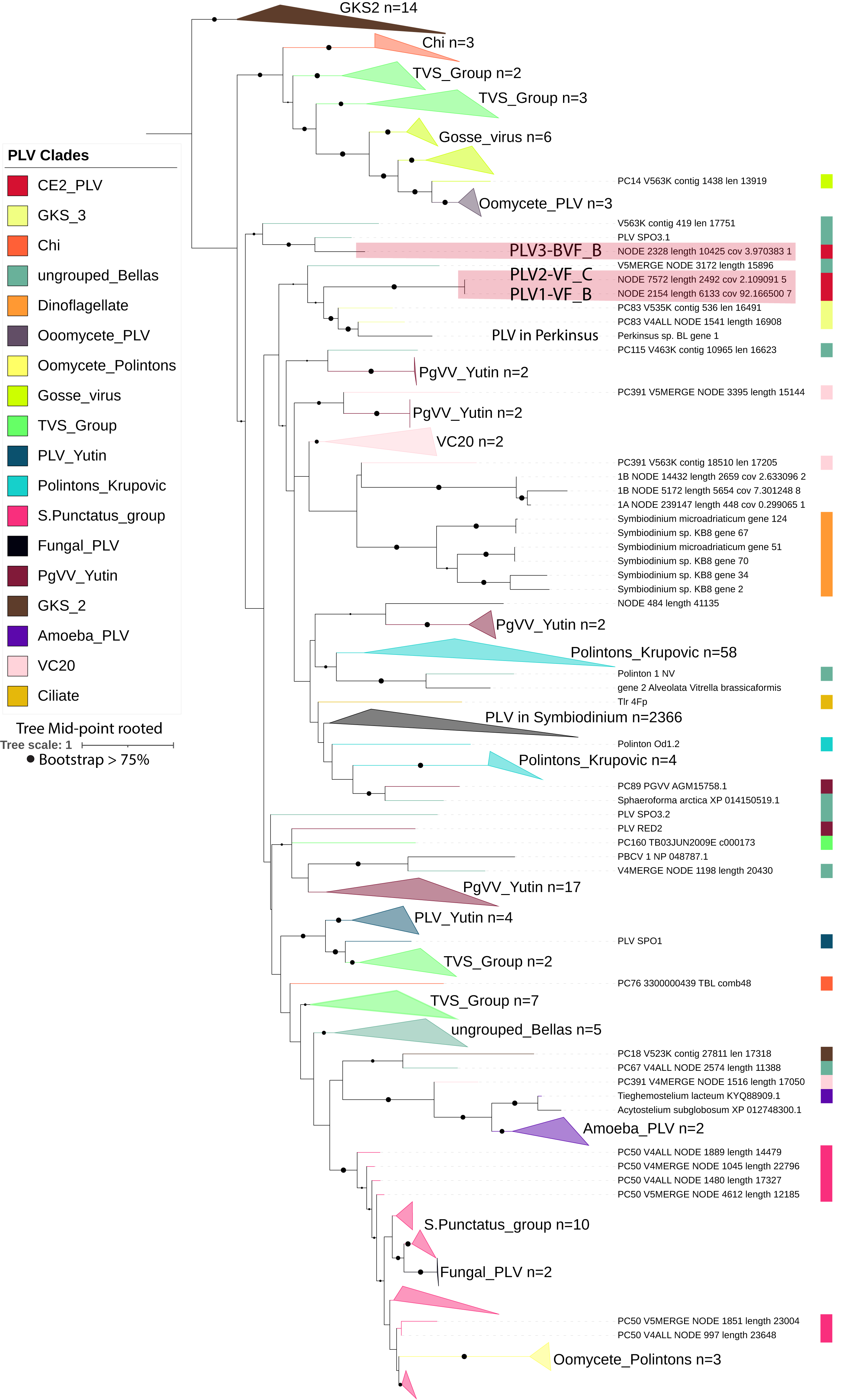

Supplement: ycag051_Supplementary_material [file ycag051_supplementary_material.zip › SupplementalFigure9.tif]

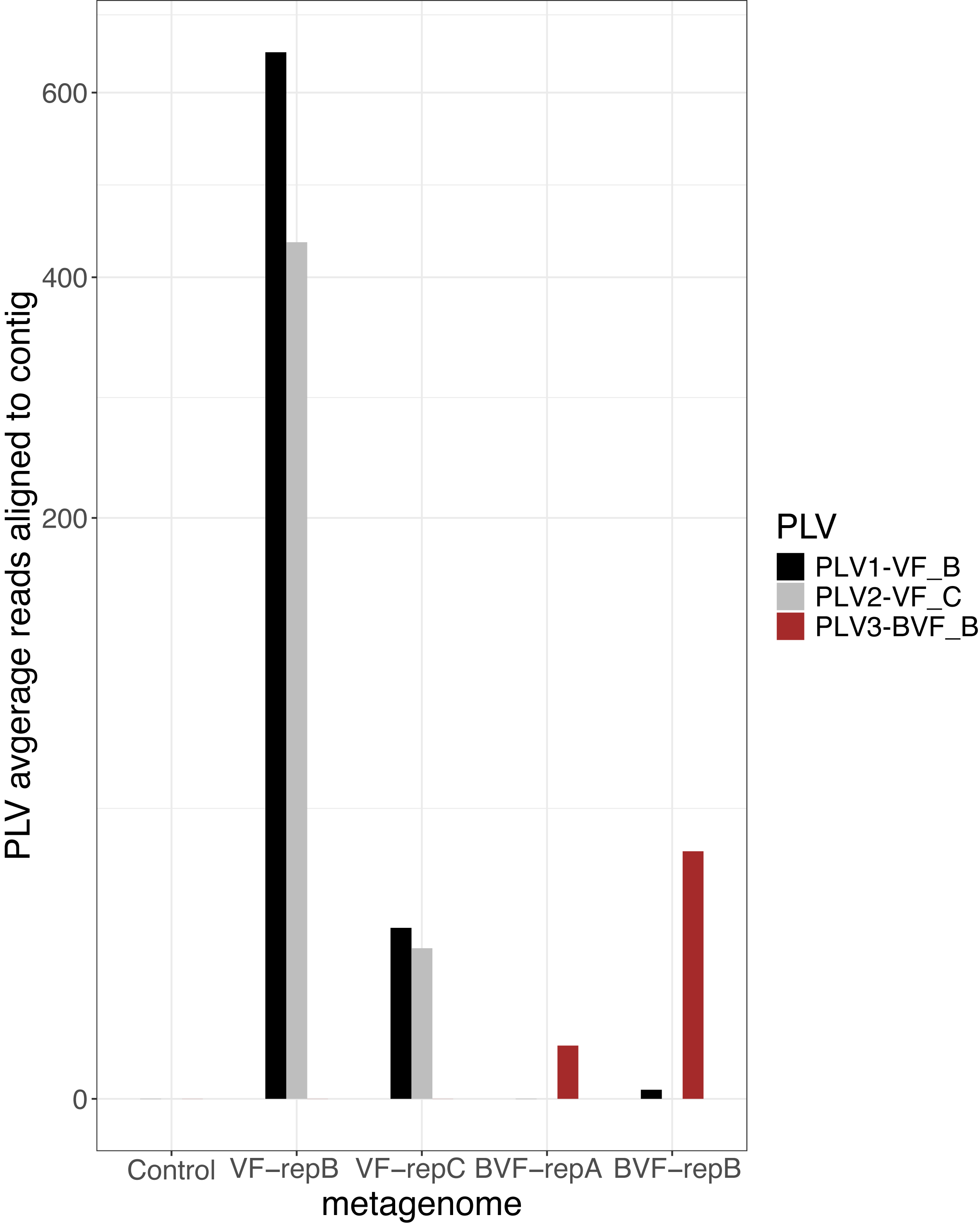

Supplement: ycag051_Supplementary_material [file ycag051_supplementary_material.zip › SupplementalFigure10.tif]
